# Characterization of the AGR2-NPM3 axis uncovers the AGR2 involvement in PD-L1 regulation in colorectal cancer

**DOI:** 10.1038/s41598-024-72990-z

**Published:** 2024-09-20

**Authors:** Andrea Martisova, Jakub Faktor, Tereza Sosolikova, Iveta Klemesova, Tamara Kolarova, Jitka Holcakova, Roman Hrstka

**Affiliations:** 1 Research Centre for Applied Molecular Oncology, Masaryk Memorial Cancer Institute, Zluty Kopec 7, Brno, 65653 Czech Republic; 2 National Centre for Biomolecular Research, Faculty of Science, Masaryk University, Kamenice 5, Brno, 62500 Czech Republic; 3 International Centre for Cancer Vaccine Science, University of Gdansk, Kladki 24, Gdansk, 80-822 Poland; 4 Department of Experimental Biology, Faculty of Science, 117204 Masaryk University, Kamenice 5, Brno, 62500 Czech Republic

**Keywords:** AGR2, NPM3, Colorectal cancer, PD-L1, Cancer models, Cancer, Cell biology, Cell signalling

## Abstract

Despite extensive research, the molecular role of AGR2 in the progression and metastasis of colorectal cancer (CRC) has not been fully characterized. We used quantitative mass spectrometry (SWATH MS) to identify differentially expressed proteins in paired CRC cell models of the SW480 and SW620 cell lines in response to AGR2 protein level manipulation. Relying on the results from SWATH MS and subsequent immunochemical validation, we selected NMP3 as the top candidate protein associated with AGR2 in CRC tumour cells in our screen. RT‒qPCR and immunochemical analysis confirmed the involvement of AGR2-mediated regulation of NPM3 at the transcriptional and posttranscriptional levels. Since PD-L1 is a constituent of the NPM3 regulatory axis, we aimed to correlate the changes in PD-L1 to the differential expression of AGR2 in our cell models. We found that AGR2 positively regulates PD-L1 levels in both SW480 and SW620 cell lines; additionally, several different CRC patient transcriptome cohorts confirmed the association of AGR2 with PD-L1. Our work reveals a new AGR2-NPM3 regulatory axis and the involvement of AGR2 in the regulation of PD-L1, which paves the way for the association of AGR2 with immune evasion in CRC cells.

**Supplementary Information:**

The online version contains supplementary material available at 10.1038/s41598-024-72990-z.

## Introduction

Colorectal cancer (CRC) is still among the top three cancer types in terms of both incidence and mortality rates worldwide. As with most cancers, the main cause of this disease is the metastatic dissemination of cancer cells to secondary sites. Metastasis is a complex multistep process involving multiple signalling pathways; however, their crosstalk and molecular mechanisms are still not fully understood. During metastasis, cancer cells acquire migratory and invasive properties, often alongside stemness, chemoresistance, and immunotherapy resistance, which promote cancer progression and evasion of treatment. Identifying the key proteins involved in this process could significantly improve therapeutic options and enable the targeted disruption of these regulatory nodes. The application of mass spectrometry (MS) in cancer biomarker research has proven to be a valuable tool in the discovery of new biomarkers. Modern MS-based proteomic methodologies provide high sensitivity, enabling the identification of low-abundance proteins across wide dynamic ranges, which is crucial for disease-specific biomarker discovery studies.

AGR2 is a member of the Protein Disulfide Isomerase (PDI) family. These proteins function as molecular chaperones in protein folding and quality control within the endoplasmic reticulum (ER)^1^. AGR2 has been implicated in tumorigenesis, with overexpression observed in multiple cancers, including prostate, lung, stomach, ovarian, pancreatic, oesophageal, and head and neck cancers^2^. Its precise role in colorectal cancer still lacks a more profound understanding, and the research thus far seems to be contradictory^3^. Our research group previously described AGR2 as a keeper of epithelial phenotype and identified a double negative feedback loop between AGR2 and ZEB1, a well-known epithelial–mesenchymal transition (EMT)-promoting transcription factor^4,5^. Apart from localising in the ER, AGR2 is also secreted extracellularly where it exhibits proinflammatory, pro-EMT, promigratory, and proangiogenic properties^6^. Recently, a new phenomenon of ER-to-cytosol signalling (ERCYS) was discovered, in which members of the ER are refluxed into the cytosol upon the induction of ER stress. During this process, AGR2 has a gain of function as a non-genetic p53 inhibitor^7^. Intracellular AGR2 contributes to proliferation, apoptosis resistance, genomic integrity, and adhesion, but the molecular mechanisms underlying these processes are still unclear^8,9^.

Nucleoplasmin-3 (NPM3), which we identified as a protein with a similar expression pattern to AGR2 in CRC, is an emerging oncoprotein primarily localised in the nucleus and nucleolus, where it exerts its function in ribosome biogenesis by regulating pre-rRNA synthesis and chromatin remodelling function when bound in a pentameric conformation with NPM1^10,11^. In lung adenocarcinoma, NPM3 is positively correlated with proliferation, while in gastric cancer, it promotes PD-L1-mediated immune escape^12,13^.

The plausible involvement of AGR2 and NPM3 in the regulation of PD-L1 expression might be of interest, as cancer cells are characterized by their ability to evade immune surveillance, during which they may either lose their antigenicity, orchestrate an immunosuppressive microenvironment or reduce their immunogenicity by expressing immunosuppressive molecules such as PD-L1^2^. The interaction between PD-L1 expressed on the cancer cell surface and PD-1 expressed on T-cells results in T-cell suppression and programmed cell death^14^. Antibodies targeting this interaction are routinely used in antitumour immunotherapy with varying degrees of success due to tumour heterogeneity^15^. Describing new targets that could help predict the extent of successful immunotherapy or aid in its effect is still highly desirable.

This study was based on whole-proteome analysis of colorectal cancer cell lines with manipulated AGR2 expression. We chose a paired CRC cell line model consisting of a primary SW480 adenocarcinoma cell line and a SW620 cell line derived from lymph node metastasis of the same tumour^16^. Using this model, we sought to elucidate additional molecular processes that might be affected by the presence or absence of AGR2. The NPM3 protein emerged as a prominent hit in our analysis; therefore, we further focused on the AGR2-NPM3 regulatory axis and its possible involvement in PD-L1 regulation.

## Results

### Quantitative mass spectrometry investigation of AGR2-associated effects on the proteome landscape in CRC cell line models

**Fig. 1:**
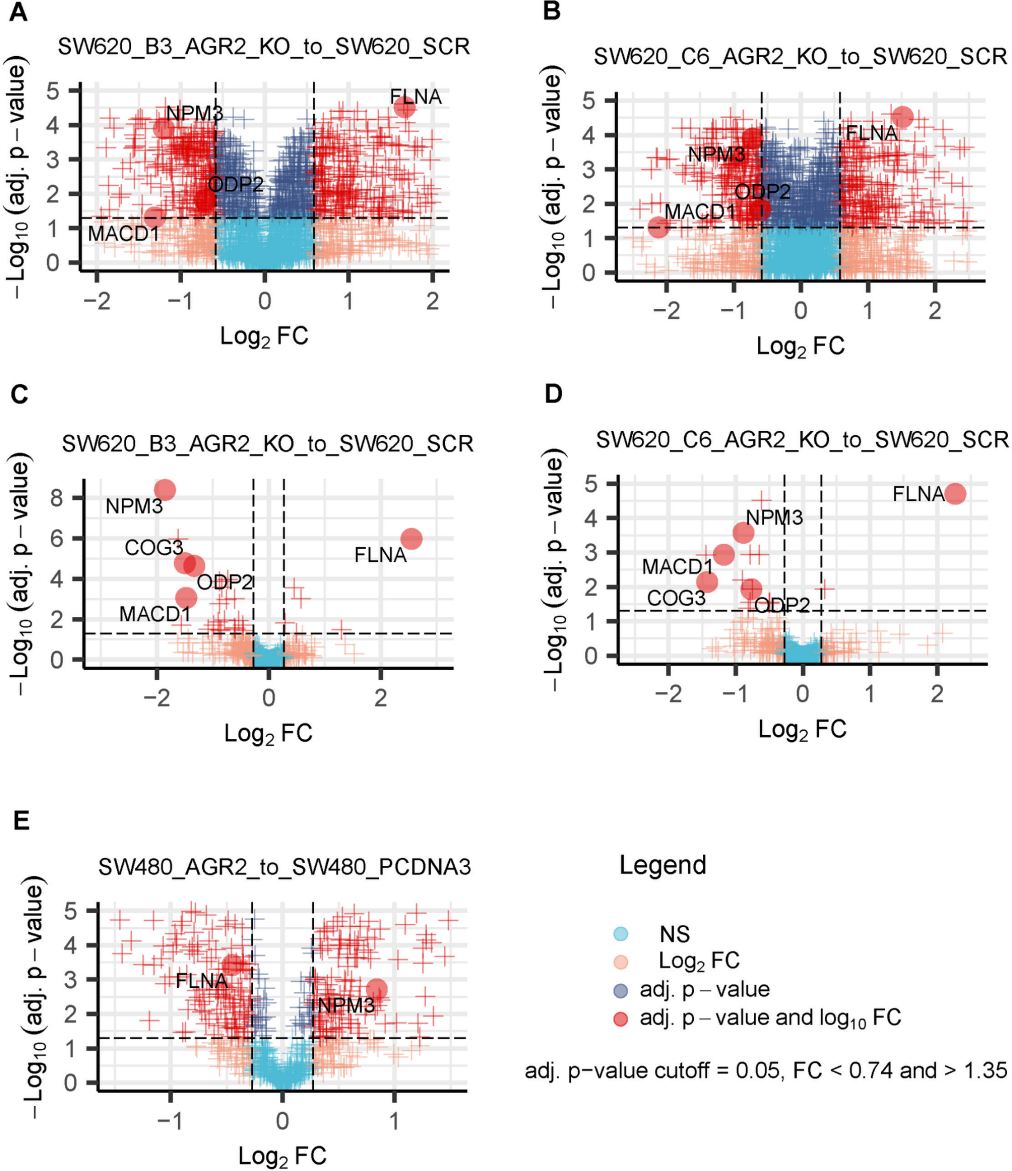
Visualisation of quantitative mass spectrometry analyses (SWATH MS) from two independent experiments performed on SW620 cells and from one experiment on SW480 cells revealing the regulation of five selected proteins in response to manipulation of the AGR2 level. Volcano plots show the significant changes in protein levels in the (**A**) AGR2 CRISPR-Cas9-mediated knockout clone SW620 KOAGR2 B3 and (**B**) SW620 KOAGR2 C6 in relation to SW620 scr. (**C**,** D**) Independent validation using the same model. (**E**) Volcano plot of differentially expressed proteins in SW480 AGR2 cells with respect to SW480 pcDNA3 cells (endogenously negative for AGR2), showing inverse regulation of FLNA and NPM3 as a response to AGR2 overexpression.

We performed SWATH MS analyses comparing SW620 scr (a negative control endogenously expressing AGR2) with two AGR2 CRISPR-Cas9-mediated knockout clones, SW620 KOAGR2 B3 and SW620 KOAGR2 C6, in two independent mass spectrometry datasets (labelled Exp 1 and Exp 2) using two distinct SWATH MS data analysis pipelines. Initially, we generated the diagnostic plots (PCA and sample correlation, Supplementary Fig. S1 A-D) revealing correlations between replicates across both datasets but also between experimental conditions, suggesting that the clonality of SW620 cells does not impose a substantial difference on protein landscapes upon AGR2 manipulation (see Supplementary Fig. S1 A and C). Diagnostic plots suggested, as expected, that SW620 scr cells are relatively more distinct from SW620 KOAGR2 clones, thus revealing an impact of AGR2 protein level on their proteomes (Supplementary Fig. S1 A). Moreover, hierarchical clustering in the heatmap from the proteomic screen of Exp 1 (Supplementary Fig. S1 C) effectively segregated SW620 scr cells from AGR2-manipulated cells. On the other hand, these effects were not observed to such an extent in the second dataset (Exp 2), suggesting no extensive effect of changing the AGR2 level on the proteome landscape (see Supplementary Fig. S1 B and D). We attribute the increased variability among replicates in the second dataset (Exp 2) to variability during sample preparation, data acquisition, data analysis and differences in cell growth conditions. Indeed, quality control (QC) on the LC-MS/MS data level revealed the differences among the datasets which is the main cause for inconsistent number of significantly changed proteins among Exp 1 and Exp 2. Supplementary Fig. S2 shows the protein intensities in both datasets. First glance on the Supplementary Fig. S2 A and S2 B suggests that Exp 2 (Supplementary Fig. S2 B) contains significantly more NA (not available/detected) or 0 values (as first quantile is for some LC-MS/MS runs close to 0) for proteins, suggesting that there will be relatively higher variability and less consistency among the conditions of Exp 2 in contrast to Exp 1 (Supplementary Fig. S2 A) which harbours significantly less NA or 0 values. Despite this, we proceeded with our analysis, relying on the assumption that genuine biological signals would prevail over the variance introduced by technical factors. Separate analyses of both datasets revealed concerted protein changes across the compared conditions, resulting in a panel of five proteins whose expression significantly changed in response to AGR2 manipulation in SW620 cells. Our selection criteria were based on the consistency of protein regulation across two datasets, two data analysis pipelines and two cell line clones, the significance of the fold change, and probable biological relevance to the AGR2 protein network. Detailed insight into a panel of significantly and consistently changed proteins demonstrated in both SW620 AGR2 knockout clones, B3 and C6, compared to the corresponding negative control (SW620 scr, endogenously expressing AGR2) in two independent experiments analysed by two data analysis pipelines is shown in Table 1; Fig. 1A, B, C, D. Three of the proteins, namely, NPM3, ODP2, and MACD1, were downregulated, while FLNA was upregulated in response to AGR2 knockout in our preliminary SWATH MS screen (Exp 1) (Table 1; Fig. 1A, B). The second mass spectrometry dataset (Exp 2) of the SW620 cell line clones confirmed the regulation of selected NPM3, ODP2, MACD1 and FLNA proteins in the same cellular models. At the same time, it suggested COG3 as an additional downregulated protein in response to AGR2 knockout (Table 1; Fig. 1C, D). The selected proteins obeyed similar trends in both analyses. Nevertheless, slight differences in both datasets, e.g., the absence of quantitation for COG3 (see Table 1) in one of the datasets, could be attributed to several commonly occurring reasons, as mentioned above, or to the diversity of algorithms included in the data processing pipeline. These findings could explain slight inconsistencies in the fold changes of the same set of proteins across independent experiments. The evidence for these statements could be supported by the observation of fewer significantly quantitated proteins in the second dataset (Exp 2) (Fig. 1C, D, Supplementary data 1), which also led to the identification of fewer proteins than in the first dataset (Exp 1) (Fig. 1A, B, Supplementary data 1). Additionally, we would like to emphasize the importance of using different data analysis pipelines, as is apparent from Table 1 and Supplementary data 1. In our case, using two distinct SWATH MS pipelines enabled the identification of consistent trends in protein regulation in response to AGR2 manipulation. Table 1 clearly suggests that relying solely on a single SWATH data analysis pipeline would substantially reduce the list of potential AGR2-responsive candidates. The differences in proteome coverage and the significance of the results among the pipelines could also be partially attributed to differences in the mass spectrometry search engines, SWATH data extraction algorithms and statistical evaluation algorithms. Therefore, we recommend that readers use “multipipeline” approaches to analyse SWATH/DIA MS data in depth^17^.

To further elucidate and confirm the general role of AGR2 on the most promising candidates in CRC, we included the biologically related SW480 cell line (derived from primary adenocarcinoma of the same patient as SW620 cells)^16^. We generated a stable AGR2-overexpressing SW480 clone, since the canonical SW480 cell line is endogenously negative for AGR2, and compared it to control cells (SW480 pcDNA3) (Table 1-Exp 3, Fig. 1E). NPM3 and FLNA were identified as significantly changed proteins in relation to AGR2. As logically expected, NPM3 was significantly upregulated, while FLNA showed a downregulated pattern. The corresponding fold changes and adjusted p-values for the selected proteins are listed in Table 1. Taken together, our proteomic screen suggests a common mechanism of AGR2 acting over FLNA and NPM3 proteins in SW480 and SW620 colorectal cancer cell models, as demonstrated by both the overexpression and silencing of AGR2. Supplementary Data 1 provides a full list of quantified proteins.

**Table 1:** Detailed insight into the regulation of five selected proteins responding to AGR2 level manipulation in two SW620 clones across two independent quantitative mass spectrometry (SWATH MS) datasets (Exp 1 and Exp 2). This regulation was partially confirmed by a third SWATH MS dataset (Exp 3) showing an inverse response to AGR2 overexpression in the AGR2-negative SW480 cell line with induced overexpression of AGR2. The table highlights the importance of using multiple cell line clones, their investigation in independent MS datasets and using multiple search engines accompanied by different SWATH data processing pipelines to select the best candidates responding to AGR2 manipulation in CRC. In addition, the table highlights the importance of SWATH mass spectrometry in general, which allows parallel acquisition of quantitative datasets for multiple proteins, potentially revealing the dominant effects of AGR2 on the proteomic landscape in CRC. This approach could elucidate the role of AGR2 and identify promising therapeutic targets or pathways involved in CRC. “N/A” indicates that the protein was not quantified due to its absence in the spectral library.

ProteinPilot-SWATH Acquisition MicroApp-MarkerView pipeline															
Protein	Exp 1: AGR2 Silencing in SW620 clones						Exp 2: AGR2 Silencing in SW620 clones						Exp 3: AGR2 overexpression in SW480 with no AGR2		
	SW620_B3_AGR2_KO_to_SW620_SCR			SW620_C6_AGR2_KO_to_SW620_SCR			SW620_B3_AGR2_KO_to_SW620_SCR			SW620_C6_AGR2_KO_to_SW620_SCR			SW480_AGR2_to_SW480_pcDNA3		
	FCH	P-val	Adj.P	FCH	P-val	Adj.P	FCH	P-val	Adj.P	FCH	P-val	Adj.P	FCH	P-val	Adj.P
COG3	N/A	N/A	N/A	N/A	N/A	N/A	N/A	N/A	N/A	N/A	N/A	N/A	N/A	N/A	N/A
FLNA	3.18	7.12E-08	3.02E-05	2.86	6.82E-06	0.00019	3.59	1.95E-06	0.00044	3.37	0.00033	0.017	0.65	6.63E-05	0.0010
MACD1	0.40	0.022	0.049	0.23	0.0086	0.022	0.43	0.0025	0.024	0.40	0.0016	0.039	0.90	0.81	0.87
NPM3	0.43	3.00E-06	0.00013	0.61	1.94E-05	0.00031	0.31	0.00030	0.0065	0.47	0.00086	0.027	1.29	0.00018	0.0017
ODP2	0.61	0.0050	0.015	0.66	0.00081	0.0037	0.40	2.41E-06	0.00049	0.67	6.52E-07	0.0012	1.58	0.32	0.43
**MSFragger-Skyline-MSstats pipeline**															
	FCH	P-val	Adj.P	FCH	P-val	Adj.P	FCH	P-val	Adj.P	FCH	P-val	Adj.P	FCH	P-val	Adj.P
COG3	N/A	N/A	N/A	N/A	N/A	N/A	0.35	1.47E-07	1.71E-05	0.37	0.00014	0.0072	N/A	N/A	N/A
FLNA	2.96	9.89E-13	2.77E-11	2.76	3.08E-12	9.12E-11	5.86	6.39E-09	1.08E-06	4.82	4.24E-08	1.97E-05	0.73	9.28E-05	0.00038
MACD1	N/A	N/A	N/A	N/A	N/A	N/A	0.36	1.91E-05	0.00089	0.44	1.50E-05	0.0012	N/A	N/A	N/A
NPM3	0.54	0.00041	0.0011	0.66	0.0073	0.0145	0.28	8.77E-12	4.10E-09	0.54	1.72E-06	0.00027	1.79	0.00057	0.0019
ODP2	N/A	N/A	N/A	N/A	N/A	N/A	0.4	2.48E-07	2.32E-05	0.59	0.00028	0.012	N/A	N/A	N/A

**Fig. 2:**
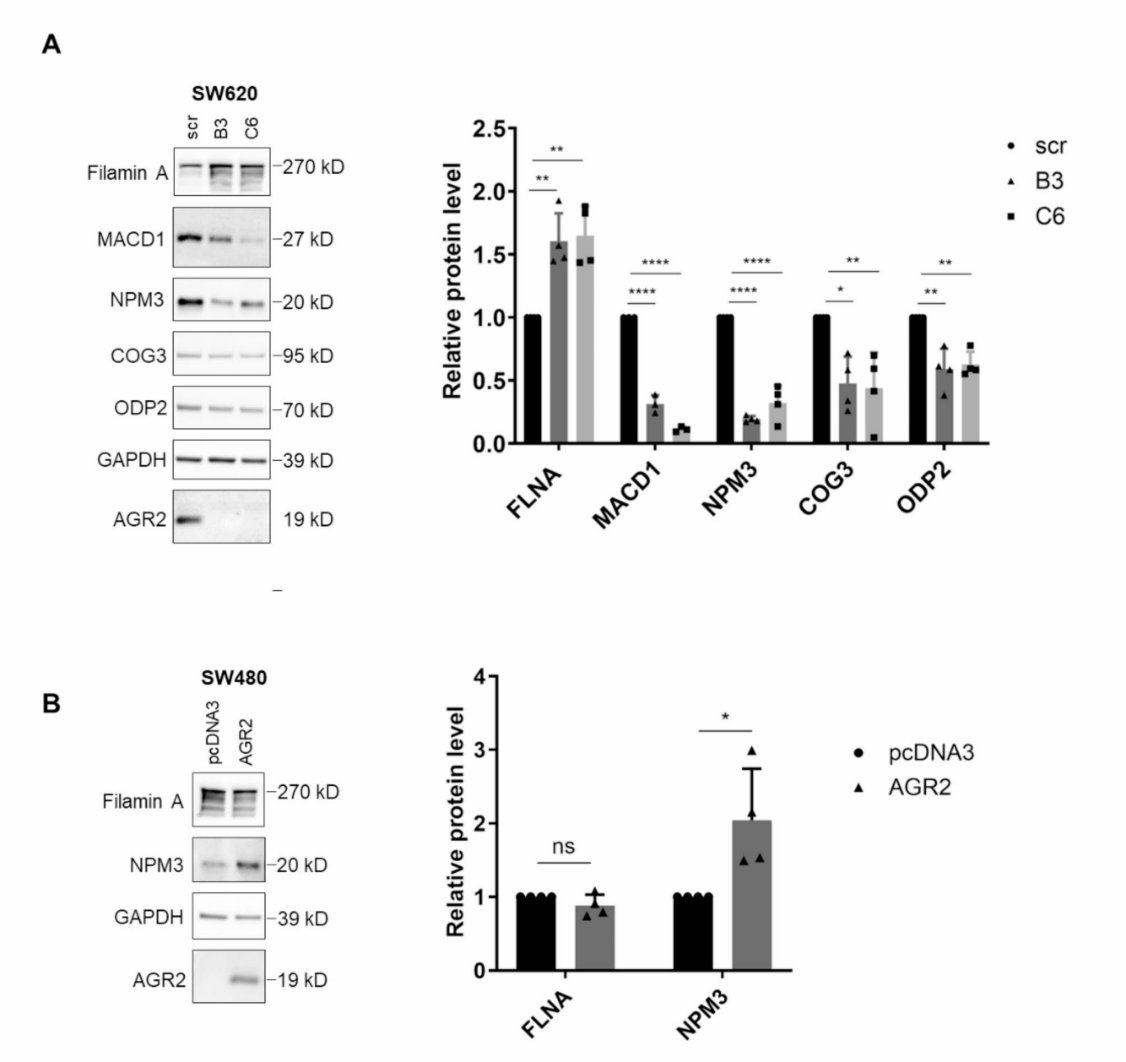
Immunochemical validation of SWATH MS data. Western blot analysis of selected proteins in (**A**) SW620 and (**B**) SW480 cells. GAPDH served as a loading control. The graphs on the right show the mean ± SD densitometry values of at least three independent biological replicates normalised to GAPDH. The asterisks indicate the significance levels determined by Ordinary One-way ANOVA or unpaired t test for SW620 and SW480 cells, respectively. The figure shows cropped blots, the originals are presented in Supplementary Fig. S3. * p 0.05, ** p 0.01, *** p 0.001, **** p 0.0001.

The panel of five protein candidates, with a central focus on NPM3 and FLNA, was further validated using the western blot technique, which compared the same conditions as our mass spectrometry screen. Consistent with the MS data, we confirmed significant changes in NPM3, FLNA, MACD1, ODP2, and COG3 levels in SW620 cells (Fig. 2A) and NPM3 level in SW480 cells (Fig. 2B).

Since NPM3 changed significantly and uniformly in all analysed clones in accordance with AGR2 expression, we selected this protein for further analysis.

#### The modulation of AGR2 expression evokes changes in NPM3 expression

**Fig. 3:**
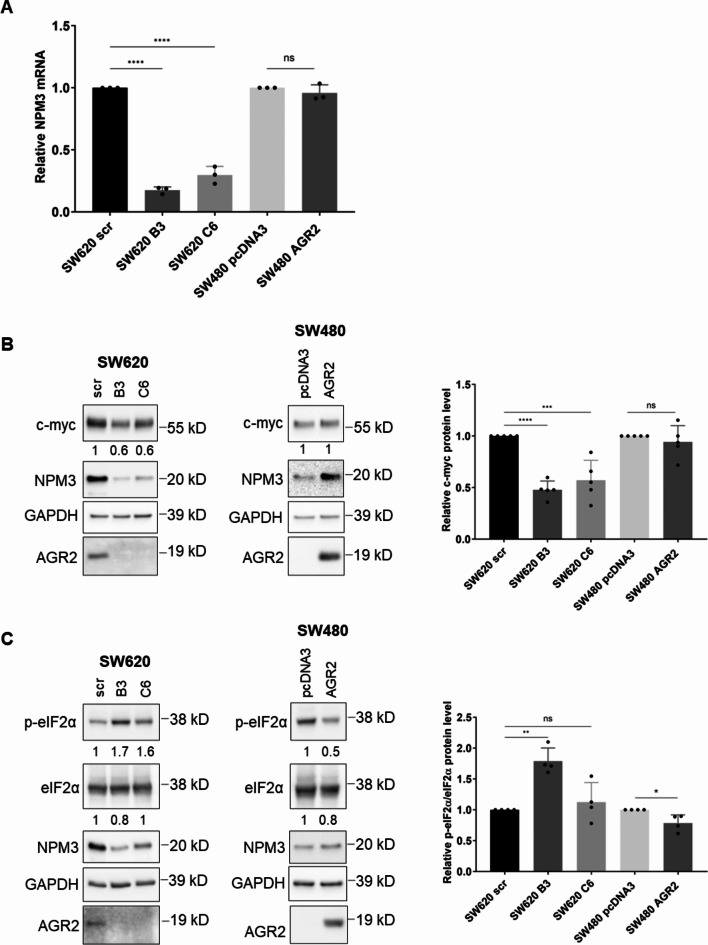
AGR2-dependent regulation of NPM3. (**A**) Analysis of NPM3 mRNA levels in SW620 and SW480 cell lines and their respective AGR2 clones. RT-qPCR was performed in three biological replicates, and the data are presented as 2^−ddCt^ mean values ± SD. GAPDH served as an endogenous control. The results for the endogenous HPRT1 control are presented in Supplementary Fig. S4 (A) (**B**) Changes in the c-myc level in SW620 and SW480 cell lines and AGR2 clones. (**C**) Analysis of eIF2α phosphorylation in AGR2 clones of SW620 and SW480 cells. The numbers represent densitometry values normalised to GAPDH loading control. The graphs on the right show the mean ± SD densitometry values of four independent biological replicates normalised to GAPDH. The level of p-eIF2α is shown as relative to the total eIF2α level. The asterisks indicate the significance levels determined by Ordinary One-way ANOVA or unpaired t test for SW620 and SW480 cells, respectively. The statistical significance is indicated by asterisks. The figure shows cropped blots, the originals are presented in Supplementary Fig.S5 A and (B) * p 0.05, ** p 0.01, *** p 0.001, **** p 0.0001.

First, we attempted to elucidate the molecular process(es) involved in the changes in NPM3 expression induced by AGR2 modulation. We analysed the mRNA levels of the NPM3 transcript in SW620 and SW480 models using RT‒qPCR (Fig. 3A). In the case of SW620 cells, NPM3 mRNA was significantly downregulated in both KOAGR2 clones compared to scr cells. In contrast, NPM3 mRNA levels were not affected by AGR2 in SW480 cells (Fig. 3A), suggesting that AGR2 modulates the regulation of NPM3 at the protein level.

Since AGR2 is neither a transcription factor nor contains any RNA binding motif, we considered several models of NPM3 mRNA level modulation by endogenous AGR2 in SW620 cells. We recently described a double negative feedback regulatory loop between AGR2 and ZEB1, a well-known EMT-driving transcription factor^4^. Since the ZEB1 binding site was identified in the NPM3 promoter, according to the Harmonizome 3.0 database, ZEB1 could serve as a repressor of NPM3 expression^18^. Nevertheless, using siRNA-mediated silencing of ZEB1 and subsequent WB detection, we did not observe any significant changes in the NPM3 protein levels (see Supplementary Fig. S4 B).

Another mechanism involved in regulating the NPM3 mRNA level would be the binding of PUM1 to NPM3 mRNA on its 3´UTR, leading to its stabilisation where AGR2 presence could presumably positively enhance this process^12^. Therefore, we analysed the rate of NPM3 mRNA decay in SW620 scr and AGR2 knockout clones and observed a slight decrease in NPM3 mRNA stability after actinomycin D treatment (Supplementary Fig. S4 D). However, these changes did not prove to be significant; therefore, we did not deem this mechanism to be a key regulator of NPM3 expression after AGR2 knockout.

A study by Ciribilli and colleagues identified NPM3 as a c-myc-regulated gene in lung papillary adenocarcinoma^19^. Indeed, a significant decrease in NPM3 mRNA levels in both SW620 AGR2 knockout clones was reflected by decreased c-myc expression, as determined by WB (Fig. 3B). In contrast, no changes in c-myc levels were observed in SW480 cells (Fig. 3B). Taken together, we propose a c-myc-dependent regulatory mechanism by which AGR2 regulates NPM3 expression at the transcriptional level in SW620 cells. This, in turn, translates into the induction of NPM3 expression at the protein level. On the other hand, this mechanism does not appear to occur in the CRC cell line SW480, which responds similarly to AGR2 modulation by changes in NPM3 protein levels.

Protein synthesis in eukaryotes is controlled by signals and stresses via a common pathway called the integrated stress response, in which the major intrinsic factor is ER stress due to the accumulation of unfolded proteins^20,21^. As AGR2 belongs to the family of protein disulphide isomerases, it has been implicated in the regulation of ER homeostasis^22^. Building on the work of Higa et al., we analysed the levels of phosphorylated eIF2α in SW480 and SW620 cells with manipulated AGR2 expression^23^. Phosphorylated eIF2α is a well-known repressor of global cap-dependent protein synthesis, which could explain the lower NPM3 levels in cells without AGR2. Indeed, we observed that if the cells lacked AGR2, they had increased p-eIF2α levels (Fig. 3C).

These findings imply that the regulation of NPM3 by AGR2 does not rely on a single mechanism but rather involves several different ones. When combined together, these findings suggest that changes in AGR2 expression trigger molecular changes that influence NPM3 expression at both the transcriptional and posttranscriptional levels.

### The AGR2-NPM3 axis induces PD-L1 expression

**Fig. 4:**
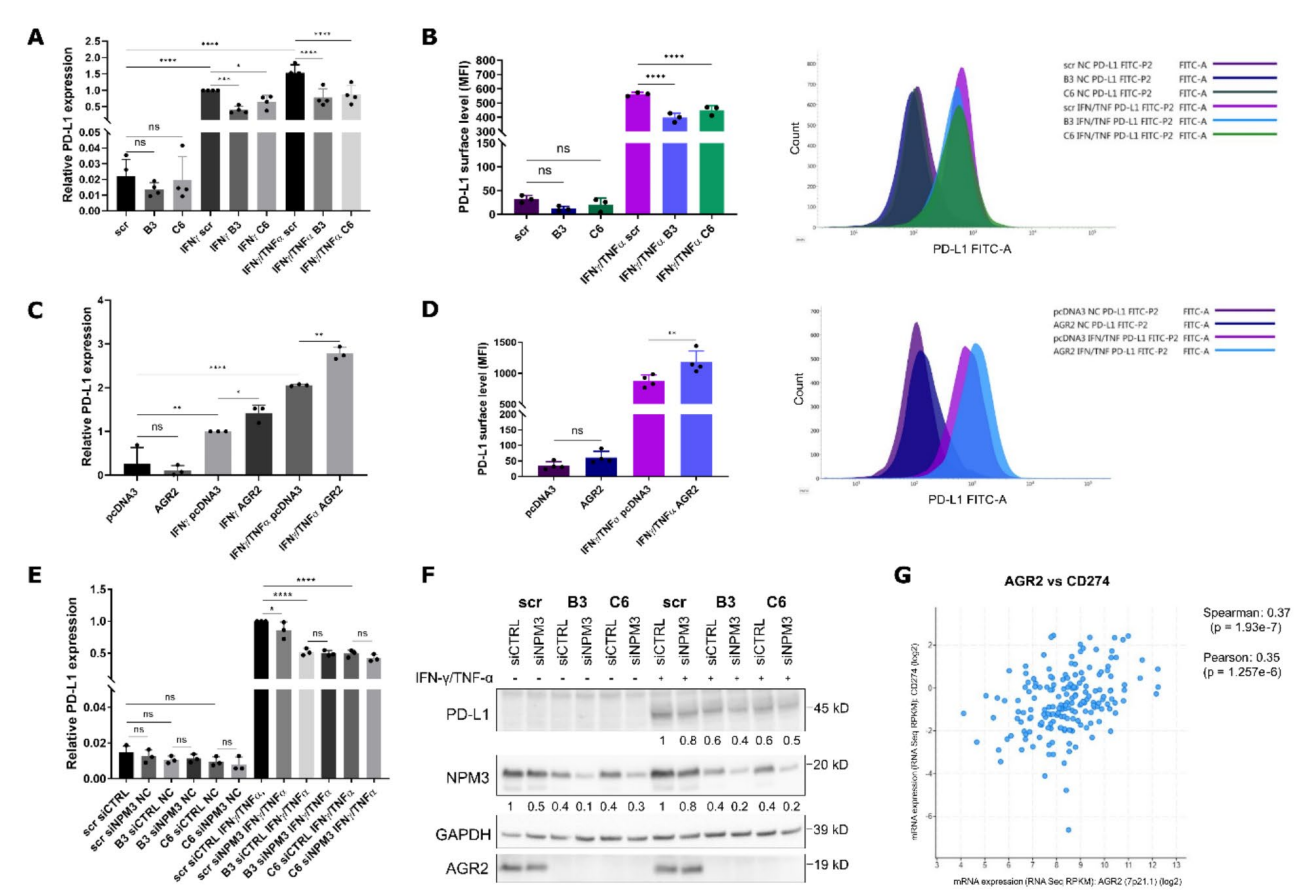
AGR2-dependent changes in PD-L1 expression. (**A**) RT‒qPCR analysis of PD-L1 mRNA levels in SW620 scr cells and its two AGR2-knockout clones B3 and C6 before and after induction with INF-γ alone or in combination with TNF-α after 4 h of treatment. The values were normalised to those of scr IFN-γ and are presented as 2^−ddCt^ mean values ± SD of four biological replicates. GAPDH served as an endogenous control. The results for HPRT1 endogenous control are presented in Supplementary Fig. S7 (A) (**B**) The surface level of PD-L1 in SW620 cells measured by flow cytometry before and after 16 h of IFN-γ/TNF-α induction. MFI values represent the median fluorescence intensity and are presented as the mean ± SD of three biological replicates. On the right is a representative histogram showing the difference in fluorescence intensities of the analysed samples. The histogram including the respective negative controls is available in Supplementary Fig. S7 (B) (**C**) PD-L1 mRNA levels in SW480 pcDNA3 and AGR2 cells treated with INF- γ or INF- γ/TNF-α after 4 h. The values were normalized to those of pcDNA3 INF- γ and are presented as 2^−ddCt^ mean values ± SD of three independent replicates. GAPDH served as an endogenous control. The results for the HPRT1 endogenous control are presented in Supplementary Fig.S7 (C) (**D**) Surface PD-L1 levels in SW480 cells are shown as the mean MFI ± SD of three independent replicates, with a representative histogram on the right. The histogram including the respective negative controls is shown in Supplementary Fig. S7 (D) (**E**) RT‒qPCR analysis of PD-L1 mRNA levels in SW620 scr cells and its two AGR2-knockout clones B3 and C6 after NPM3 siRNA silencing and 4 h INF-γ/ TNF-α treatment. The values were normalised to those of scr siCTRL INF-γ/ TNF-α and are presented as 2^−ddCt^ mean values ± SD of three biological replicates. GAPDH served as an endogenous control. The results for HPRT1 endogenous control are presented in Supplementary Fig. S7 (E) (**F**) Representative WB showing the changes in PD-L1 protein levels in SW620 clones and control cells after siRNA-mediated NPM3 and 16 h of INF- γ/TNF-α induction. GAPDH served as a loading control. The graph showing mean ± SD densitometry is presented in Supplementary Fig. S7 (F) The figure shows cropped blots, the originals are presented in Supplementary Fig. S7 (G) * p 0.05, ** p 0.01, *** p 0.001, **** p 0.0001. (**G**) Correlation analysis of AGR2 and PD-L1 expression in colorectal patient samples represented by Pearson and Spearman correlation coefficients extracted from the cBioPortal database.

Since NPM3 was recently described to be involved in regulating PD-L1 expression in gastric cancer through binding to NPM1 and promoting its binding to the PD-L1 promoter, we analysed PD-L1 expression at the mRNA level. However, our SW620 cellular model endogenously expresses low levels of PD-L1 mRNA, and without induction, we observed only a nonsignificant trend indicating the downregulation of PD-L1 in KOAGR2 cells. After the treatment with INF-γ or a combination of INF-γ and TNF-α, we observed significant induction in PD-L1 mRNA when compared to the untreated cells. Meanwhile, KOAGR2 clones expressing lower levels of NPM3 exhibited significantly decreased induction of PD-L1 compared to scr control cells (Fig. 4A).

The primary role of PD-L1 as a ligand of PD-1 is on the cell surface; therefore, we analysed the PD-L1 surface level using flow cytometry. Without INF-γ/TNF-α induction, there were only minor changes in the PD-L1 levels, with the signal being close to that of the negative control. This is of no surprise since, as mentioned above, SW620 cells have low PD-L1 expression levels without induction. After induction, we observed a significantly lower PD-L1 signal in the KOAGR2 clones when compared to the induced scr control (Fig. 4B).

The same effect was also observed in the SW480 cells, where the AGR2-positive clone had a higher level of PD-L1 mRNA after induction compared to the AGR2-negative SW480 pcDNA3 cells (Fig. 4C). This is again in accordance with the observed changes in NPM3. Additionally, the surface level of PD-L1 was consistent with our hypothesis, as we detected a significantly higher signal for SW480 AGR2 cells when compared to their AGR2-negative counterpart pcDNA3 after induction with INF-γ/TNF-α (Fig. 4D).

However, INF-γ acts on PD-L1 primarily through JAK/STAT1/IRF1 signalling^24^ while TNF-α acts mainly through NF-κB pathway, with IFN-γ also influencing this pathway^25^. Therefore, the regulation of PD-L1 expression by AGR2 during INF-γ/TNF-α presence might be independent of the NPM3 regulation. Hence, PD-L1 and NPM3 mRNA and protein levels were analysed by RT-qPCR (Fig. 4E) and WB (Fig. 4F) in cells transiently transfected by siRNA against NPM3 with or without INF‑γ/TNF-α induction. Without induction, PD-L1 mRNA levels were very low, and PD-L1 protein was not detectable by WB analysis. After induction with INF-γ/TNF-α, we observed increased PD-L1 on both mRNA and protein levels. Samples with silenced NPM3 showed decreased levels of PD-L1 on both mRNA and protein levels when compared to their respective control siRNAs (Fig. 4E, F), supporting AGR2-dependent modulation of PD-L1 levels through regulation of NPM3. However, these small changes, point towards additional regulatory mechanisms responsible for the observed differences between AGR2-positive and AGR2-negative cells.

To further support our findings of the involvement of AGR2 in PD-L1 regulation, we screened the publicly available cBioPortal database^26–28^. Our first choice was a publicly available dataset, “The Cancer Genome Atlas (TCGA) Colorectal Cancer project”, and we found that AGR2 and PD-L1 are significantly coexpressed in CRC tissue, as indicated by Spearman and Pearson correlation coefficients (Fig. 4G)^29^. In addition, we analysed other available CRC datasets in cBioPortal. We confirmed a significant correlation in mRNA levels between AGR2 and CD274 in the GDAC Firehose dataset (previously known as TCGA provisional, see Supplementary Fig. S6 A) as well as in Colorectal Adenocarcinoma TCGA PanCancer Atlas consisting of 526 samples/patients (Supplementary Fig. S6 B). Importantly, in a very recent dataset generated by whole exome and transcriptome sequencing of 348 Colon Cancers, we also found a significant correlation between AGR2 and CD274 mRNA expression (Supplementary Fig. S6 C)^30^.

## Discussion

AGR2 has gained substantial attention in cancer-associated processes and is one of the most studied proteins from the PDI family in relation to cancer. It is largely overexpressed in tumour cells when compared to their healthy counterparts. Despite the available research, its precise molecular mechanisms, especially the role of AGR2 in colorectal cancer, remain to be elucidated. Therefore, focusing on the AGR2-associated proteome changes in CRC could unveil novel regulatory mechanisms, therapeutic targets, and provide new diagnostic or therapeutic strategies.

In this work, we analysed the impact of AGR2 protein level manipulation on the proteomic landscape in a paired CRC cellular model using label-free quantitative mass spectrometry (SWATH MS). The preliminary screen of the CRISPR/Cas9-mediated AGR2 knockout clones derived from SW620 cells identified a panel of 4 significantly changed proteins (FLNA, macroD1, NPM3, and ODP2), which was confirmed in the follow-up MS analysis of independent biological replicates, which added another significantly changed protein, COG3, into the panel. Additional analysis of parental SW480 cells stably transfected to express AGR2 confirmed NPM3 and FLNA proteins as significantly changed in relation to the presence or absence of AGR2 expression. Even though we observed consistent changes in the case of NPM3 and FLNA, other proteins identified in SW620 cells were not confirmed in SW480 cells. This might be explained by the fact that the knockin of AGR2 in SW480 has a weaker effect on cells than the knockout. Additionally, the molecular background of SW620 cells may be influenced by the temporal evolution of the tumour cells and their adaptation to the environment since SW620 is a metastatic cell line that was derived a year later from a metastasis arising from the primary tumour represented by SW480 cells^15^.

Nevertheless, for the first time, we identified the regulatory dependence of NPM3 and FLNA on AGR2 protein expression level in two analysed CRC cell lines. Taken together, our results are supported by consistent findings across multiple levels, AGR2 dependence of NPM3 and FLNA was demonstrated in two knockout clones and two independent analyses, while inverse regulation was observed with AGR2 overexpression. Moreover, we showed that AGR2 can regulate NPM3 expression at both transcriptional and translational levels in CRC cells, depending on the cellular and genetic background of these malignancies. Looking deeper into the potential regulatory mechanism, NPM3 was identified as a c-myc target gene^19^. Additionally, AGR2 silencing inhibited c-myc levels in breast cancer cells, as shown by Vanderlaag et al.^31^. Therefore, we proposed a potential mechanism by which reduced AGR2 expression might affect NPM3 levels by decreasing c-myc protein level. However, this regulatory mechanism was not observed in SW480 clones, indicating the presence of additional AGR2-dependent mechanism(s) involved in the regulation of NPM3 expression. AGR2, one of the PDI members, has a role in ER stress regulation, and its downregulation affects ER homeostasis. Even siRNA-mediated silencing of AGR2 resulted in increased phosphorylation of eIF2α in HeLa cells^23^. Therefore, we analysed the p-eIF2α levels in our cellular models with manipulated AGR2 expression. Both showed increased phosphorylation levels if AGR2 was not present. Since the phosphorylation of eIF2α is a repressor of cap-dependent protein synthesis, it is possible that the absence of AGR2 is directly linked to this phenomenon and thus represents a key prerequisite for the decrease in the NPM3 level.

NPM3 is emerging as a significant player in tumorigenesis and cancer progression. The limited number of publications available at this moment hints at its main roles in regulating ribosome biogenesis, enhancing activator-dependent transcription, and improving immunoregulatory processes through its interaction with NPM1^11,12,32^. Pancancer analysis revealed increased levels of NPM3 in various cancer types, including CRC, compared to healthy tissues^33^.

A recently published article showed that NPM3 mRNA is stabilised by PUM1 binding and that the NPM3 protein subsequently binds to and enhances the translocation of NPM1 into the nucleus, where NPM1 serves as an inducer of PD-L1 expression^12,34^. While NPM1 was shown to directly bind to the PD-L1 promoter NPM3 was not. Rather, it seems that the binding of NPM3 to NPM1 could influence chromatin organisation, favouring transcriptional activation^12^. We did not observe any direct significant association between AGR2 expression and the PUM1 regulatory mechanism of NPM3 expression. However, we indeed observed a positive correlation between AGR2 and PD-L1 expression compared to that in AGR2-negative cells. The main limitation of our system is that the analysed cell lines do not endogenously express high levels of the PD-L1 protein, in contrast to the ones from the study of Wang and colleagues^12^. PD-L1 might be either expressed constitutively or induced by IFN-γ and other cytokines produced by immune cells^24,35^. Therefore, in order to study PD-L1 expression, we treated cells with IFN-γ alone or in combination with TNF-α, as IFN-γ and TNF-α are known to synergise and cooperatively enhance the expression of a number of genes involved in inflammation^36,37^. We are aware that the induction of PD-L1 by IFN-γ and TNF-α signalling is mainly mediated by the JAK-STAT1/3 and NF-κB pathways; however, the study by Qin et al. showed that the regulation of PD-L1 expression by NPM1 may be relevant even in the presence of IFN-γ^24,35^. More precisely, the authors showed that silencing of NAT10, an acetyltransferase needed for the role of NPM1 as a PD-L1 inducer, leads to decreased PD-L1 signals during IFN-γ induction. Therefore, the whole machinery composed of AGR2-NPM3-NPM1 could induce PD-L1 alongside or with the IFN-γ signalling cascade. Interestingly, NAT10 was also identified in our MS screen as downregulated in both B3 and C6 SW620 clones (Exp1), while being upregulated in SW480 AGR2. Therefore, to support our claim, we analysed PD-L1 expression in our SW620 system after siRNA-mediated silencing of NPM3. Indeed, we observed a slight decrease in the PD-L1 signal compared to that in control cells. However, the downregulation of PD-L1 was more profound in KOAGR2 cells, most likely due to already significantly decreased NPM3 and the complete absence of AGR2, which could affect PD-L1 through additional regulatory mechanism(s). For instance, our SWATH screen identified other regulators of NF-κB pathway as significantly changed upon AGR2 knockout. TNF-α binds to TNFR while IFN-γ to IFNR but both cytokines may activate the NF-κB signalling pathway which results in induced PD-L1 transcription^25^. We identified CSNK2A1 and CSNK2B, subunits of casein kinase (CK2), as significantly downregulated in AGR2 knockout clones. CK2 enhances NF-κB signalling by phosphorylation of IκBα (inhibitor of NF-κB) which leads to IκBα degradation while simultaneously phosphorylating p65 subunit of NF-κB which results in an increase of NF-κB transcriptional activity^38^. In parallel, we also identified COMMD6 as upregulated in AGR2 knockout clones. COMMD6 inhibits TNF-induced NF-κB activation^39^. Therefore, we presume that AGR2 knockout may affect PD-L1 expression also by additional mechanisms than just through the NPM3 axis. Additionally, the positive correlation between AGR2 and PD-L1 in CRC is also supported by cohorts available in the cBioPortal database, indicating the overlap of our findings with data from CRC patient samples.

The importance of PD-L1 has been extensively studied as a ligand of PD-1, resulting in dampening of immune responses^40^. PD-1 is mainly expressed in T-cells, B-cells, NK cells, and MDSCs, while PD-L1 is found on antigen-presenting cells and tumour cells. The interaction between PD-1 and PD-L1 leads to the inhibition of their cytotoxic activity and the transformation of effector T-cells into Tregs, resulting in immunosuppression^41^. Therefore, blocking this interaction is a vital immunotherapy target. However, only a limited number of CRC patients respond well to PD-1/PD-L1 monotherapy^42–44^.

The molecular classification of CRC that considers the tumour microenvironment (TME) includes four categories, where CMS1 (consensus molecular subtype) comprises tumours with mismatch repair deficiency (dMMR) responsible for microsatellite instability (MSI-H), which results in increased tumour mutational burden (TMB). This results in the production of aberrant proteins that are presented as neoantigens, driving the infiltration of immune cells and resulting in so-called “immune hot” phenotypes^45^. These tumours also express elevated PD-L1, which is mostly induced by INF-γ and other cytokines produced by tumour-infiltrating lymphocytes, to dampen the immune response. It is these tumours that respond well to PD-1/PD-L1 therapy. Interestingly, AGR2 was shown to be expressed in CRC samples with different aetiologies, including MSI-H tumours^46,47^. However, they represent only approximately 15% of all CRC cases^44,48^. CMS2 and CMS3 are poorly immunogenic and labelled as “immune cold” tumours. Lastly, CMS4 is a category characterised as poorly differentiated with mesenchymal traits and immunosuppressive TGF‑β signalling with high heterogeneity for immune cell infiltration, meaning they may be immune cold or immune hot, but the majority of patients exhibit an immune cold phenotype^49^. In practice, patients may belong to more than one category described above. Our analysed cell lines belong to CMS4 and are labelled as MSS^50^. Therefore, they are a great representative of the worst prognosis for CRC with most probably immune cold TME and unresponsive to PD-1/PD-L1 blockage as there are no immune cells available to elicit an immune response.

Possible ways to transform immune-cold to immune-hot tumours that could benefit from PD‑1/PD-L1 blockage include combining multiple treatments. Chemotherapy is known to induce immunogenic cell death, promote CD8 + cytotoxic lymphocyte infiltration, and restrict immunosuppressive cells, hence modulating the TME in favour of an immune-hot phenotype^51^. Additionally, VEGF, TGF-β, or IL-10 inhibition positively affects immune cell infiltration^52–54^. Infiltrating lymphocytes and other immune cells may then secrete INF-γ, TNF-α, or interleukins that stimulate PD-L1 expression^2^.

Therefore, the inhibition of TGF-β and standard chemotherapy treatment in immune cold tumours could reduce immune evasion and favour immune cell infiltration. As AGR2 is known as a TGF-β susceptible gene, the inhibition of TGF-β would result in its upregulation, which in turn could positively enhance PD-L1 expression, hence providing another line of evidence for profitable treatment with PD-1/PD-L1 inhibitors^5^. Interestingly, a randomised phase 2 trial was performed on a combination of PD-1 antibody, HDAC inhibitor, and VEGF antibody in MSS chemotherapy refractory MMR-proficient tumours, showing promising results and providing a basis for the importance of combinatorial treatment involving PD-1/PD-L1 blockers in MSS CRC tumours^55^.

In conclusion, cancer cells accumulate abnormalities at various stages of their development that may affect the outcome of antitumour therapies. Therefore, elucidation of the regulatory processes responsible for cancer characteristics and TME status is highly desirable. In this work, we described the positive regulation of NPM3 by AGR2, which led us to the identification of the involvement of AGR2 signalling in the regulation of PD-L1 expression. Our data hints on the regulation of PD-L1 by AGR2 through the NPM3-NPM1 regulatory pathway; however, the full extent of the AGR2 mechanism of PD-L1 regulation remains to be elucidated.

## Methods

### Cell culture

SW480 and SW620 cell lines were obtained from the ATCC. All cell lines were maintained in high glucose Dulbecco’s Modified Eagle’s Medium (DMEM, Sigma-Aldrich, St. Louis, MO, USA) supplemented with 10% foetal bovine serum (FBS) (Sigma-Aldrich, USA), 1 mM pyruvate (Invitrogen, USA), and 1% Penicillin-Streptomycin Solution 100× (biosera, France). Unless otherwise stated, cells were grown to 70–80% confluence prior to treatment.

AGR2-knockout (KOAGR2) SW620 cell line was prepared using CRISPR/Cas9 technology. The guide RNA oligonucleotide (5′-AGAGATACCACAGTCAAACC-3′) that targets exon 2 of the human AGR2 gene (ENSE00003623642) was designed using Tools for Guide Design (zlab.bio/guide-design-resources). The guide RNA specifically targeted mRNA coding 21–27 aa of the AGR2 N-terminal region, which is important for AGR2 protein-mediated cell adhesion. The GFP-scrambled sequence (5′-AACAGTCGCGTTTGCGACTGG-3′) served as a control^56^. Both sequences were cloned into a LentiCRISPR-v2 vector (cat. no. 52961; Addgene) using Esp3I restriction cloning. SW620 cells (1,000,000 cells) were transfected with LentiCRISPR-v2_AGR2 or LentiCRISPR-v2_scrambled (scr). After 2 days, the cells were exposed to puromycin (2 µg/ml) and the pool of resistant cells was sorted and seeded as single colonies in 96-well plates. KOAGR2 cells were tested for AGR2 expression using western blotting. Two clones with an undetectable expression of AGR2, SW620 KOAGR2 B3 and C6, were selected for further experiments.

The SW480 cell line with stable AGR2 expression was prepared using pcDNA3 plasmid containing AGR2 coding sequence as previously described in^57^.

The siRNA-mediated silencing was performed using ON-TARGETplus™ siRNA obtained from Dharmacon (Horizon Discovery, UK). ZEB1 silencing was performed by SMARTPool of ON-TARGETplus Human ZEB1 siRNA and NPM3 silencing by SMARTPool of ON-TARGETplus Human NPM3 siRNA. The respective siRNAs were delivered into SW620 cells using Lipofectamine RNAiMax (Thermo Fisher, USA).

Cells were treated to a final concentration of 10 ng/ml TNF-α (PeproTech), 100 ng/ml IFN-γ (PeproTech), and 10 µg/ml actinomycin D (Sigma-Aldrich, USA).

### Sample preparation for mass spectrometry

Samples were lysed in 8 M urea, 0.1 M Tris (pH 8.5) (Urea buffer) with protease inhibitors (Roche, CH) and needle sonicated (VibraCell, Sonics & Materials, Inc., USA) 4 × 3 s. Protein concentration was measured using the RC-DC kit (Bio-Rad, USA) according to the manufacturer’s instructions. Approximately 100 µg of protein samples were processed via Filter-aided sample preparation (FASP) to generate tryptic peptides^58^. The protein sample was loaded onto Microcon 10 kDa cut-off filter column (Merck, Germany). Afterwards, samples were centrifuged at 14,000 g for 20 min at RT. Filter membranes were incubated with 100 µl of 5 mM tris(2-carboxyethyl)phosphine TCEP in Urea buffer for 30 min at 37 °C with agitation of 600 RPM on a thermomixer followed by subsequent centrifugation at 14,000 g for 15 min at RT. Afterwards, 20 µl of 300 mM iodoacetamide (IAA) in 100 µl Urea buffer was added and incubated for 1 min, 25 °C with the agitation of 600 RPM on the thermomixer, followed by 20 min incubation at RT in the dark. Samples were centrifuged at 14,000 × g for 20 min at RT. The cut-off filters were washed twice with 100 µl of 100 mM NH_4_HCO_3_. Filter membranes were transferred into clean microtubes. Following, 100 µl of 100 mM NH_4_HCO_3_ containing 1 µg of trypsin (AB-SCIEX, USA) was added onto the cut-off filters and left overnight at 37 °C. The next day, samples were centrifuged at 17,000 g for 20 min at RT, followed by a second peptide elution of 50 µl by 0.5 M NaCl. Desalting of peptide digests was performed using C18 desalting microcolumns MicroSpin (Harvard Apparatus, USA) inspired by Bouchal et al.^59^. Microcolumns were washed twice with 200 µl acetonitrile (AcN) with 0.1% FA (Formic acid). Column equilibration was performed by 0.1% FA in water for 15 min. Following, sample digests were loaded onto the MicroSpin columns and centrifuged at 500 g, 3 min. Columns were washed three times with 200 µl of 0.1% FA in water *(v/v)*. Afterwards, columns were transferred into clean microtubes, and peptides were first eluted with 200 µl 0.1% FA in 50% AcN in water *(v/v/v)* and 200 µl of 0.1% FA in 80% AcN in water *(v/v/v)*. The last peptide elution was performed using 200 µl of 0.1% FA in 100% AcN. Cleaned tryptic peptide samples were dried using SpeedVac. The samples were resuspended in 50 µl of 0.05% TFA (trifluoroacetic acid) and 5% AcN in water *(v/v/v)* vortexed and then sonicated for 5 min prior to LC-MS/MS. Concentration was measured using NanoDrop 2000c (Thermo Scientific, USA) at 220 nm and 280 nm. All samples were then diluted to a uniform concentration of 1 µg/µl. Approximately one microgram of peptides from each sample was used per injection, and 3 µl of each sample were pooled to prepare a representative pooled sample for spectral library generation.

### Quality control (QC) of the samples

QC of samples for mass spectrometry was initially performed using RC-DC assay (Biorad, CA, USA) which determines the presence of protein in the sample. Later the detected protein concentrations were used to normalize the amount of protein loaded to the FASP filter unit for digestion. We used 100 µg of protein for digestion. After digestion peptide samples were evaporated and dissolved in loading buffer (2.5% AcN, 0.08% TFA in water) followed by another round of QC performed on Nanodrop (Thermo, USA) to determine whether peptides were yielded. Nanodrop absorbances at 220 and 280 nm are later used to load uniform peptide amount to LC-MS system. The main QC was performed from the LC-MS data. We used several different techniques to perform QC from LC-MS/MS data such as plotting the protein intensities where we compared median intensity, quantile intensities, diagnostic protein heatmap, sample correlation heatmap and PCA (principal component analysis.

### Spectral library measurement and SWATH MS data acquisition

Four and three replicates were prepared from each cell line and were measured on LC-MS/MS system. The separation of peptides was performed using the reverse phase liquid chromatography Eksigent Ekspert nanoLC 400 (SCIEX, USA), which was connected online to the TripleTOF 5600 + mass spectrometer (SCIEX, USA). Loaded peptides were concentrated on a cartridge column with 300 mm inner diameter and 5 mm length packed with C18 PepMap 100 sorbent with 5 μm particle size (Thermo Scientific, USA) using an isocratic flow of 0.05% TFA and 5% AcN in water *(v/v/v).* Peptides were separated on a 25 cm fused-silica emitter column with 75 μm inner diameter (New Objective, USA) packed in-house with ProntoSIL C18 AQ 3 μm beads (Bischoff Analysentechnik GmbH, Germany). Separation was performed by a linear increase of 0.1% FA in AcN (solvent B) over 0.1% FA in water (solvent A) throughout 120 min effective gradient. Peptide elution started at 5% B followed by a linear increase up to 40% B during 120 min at a flow rate 300 nl/min in both DDA and SWATH experiments. The separated peptides were ionised in the nanoelectrospray. The sample for a spectral library was measured in IDA (Information dependent acquisition) mode as described in Faktor and Bouchal, 2016^60^. Briefly, the full scan was set to cover a m/z range from 400 Th to 1200 Th and following, in the MS/MS mode, the top 20 most intensive precursors were selected and fragmented during each cycle. The time for precursor exclusion was set at 12 s. Precursor ions with intensity under 50 cps were excluded from the measurement.

SWATH data acquisition method was inspired by Bouchal et al.^61^. SWATH data were measured in positive mode at m/z 400–1200 Th. This spectral set was divided into 67 precursor windows of 12 Th with 1 Th overlap. The accumulation time was set at 50 ms. The cycle time was 3.5 s. Produced ions were measured at m/z 360–1360 Th.

### SWATH data analysis using ProteinPilot-SWATH Acquisition MicroApp-MarkerView pipeline

The spectral library was developed following the manufacturers recommendations in ProteinPilot (SCIEX, USA) by searching the measured MS and MS/MS data against *Homo sapiens* database downloaded from the UniProt. The spectral library was developed from 2000 proteins identified (FDR 1%) in IDA run of pooled samples. Carbamidomethyl was set as fixed modification of cysteine and Trypsin was set as protease. Other settings in ProteinPilot were left default. Afterwards, data analysis was performed in Peakview 1.2 software (SCIEX, USA) with SWATH Acquisition MicroApp 1.0 extracting quantitative SWATH data based on the constructed spectral library. The retention window was set to 8 min. Extracted data were analysed in MarkerView (SCIEX, USA) according to the manufacturer’s recommendations. Briefly, the protein intensities were calculated by summing areas of extracted peaks of proteotypic peptides. Zero values were substituted by 1, data normalisation on total ion current was selected and finally a t-test was performed on normalised data. The final results showcase a relative difference in protein levels in selected comparisons accompanied with p-values and Benjamini-Hochberg adjusted p-values calculated using Python statsmodels.stats.multitest module.

### SWATH data analysis using MSFragger-Skyline -MSstats pipeline

IDA files were converted in AB SCIEX MS Data Converter 1.2 from .wiff into .mzML format. All searches were done against *Homo Sapiens* SwissProt + UniProt search database and reverse decoy database in MSFragger 3.4 search in FragPipe (v.15)^62^. Reverse decoy database contained equal amount of sequences as the target search database. Precursor mass tolerance was set +-25 ppm and 25 ppm was set for fragment mass tolerance. Protease was set to trypsin and carbamidomethyl was set as a fixed modification. Methionine oxidation, protein N-term acetylation were set as variable modifications. Data recalibration function was selected and selected parameters for mass tolerances were automatically optimized. Resulting .pepXML files were imported into Skyline-daily (64-bit, 20.1.9.234) and transformed to a spectral library with a 0.99 cutoff set on PeptideProphet probability^63^. Protein intensities of transitions were extracted against created spectral library from SWATH MS datafiles. No modifications were considered for the quantitative experiment. Exclusively y and b product ions with + 1 and + 2 charges were considered in peakgroup transitions. Only peakgroups that had at least 3 product ions were kept in analysis, while if more product ions were available in the spectral library, up to the 6 most intense were considered. Auto detection function of SWATH window isolation scheme from raw SWATH files was selected. Equal number of reverse decoy transitions were included in the Skyline target panel. Embedded mProphet peak scoring model was activated to determine q-values of extracted target peakgroups relying on decoy peakgroups^64^.

Statistical analysis was executed in MSstats package running under R (version 4.0.0)^65^. mProphet peakgroup q-cut-off was set to q-value < 0.01. Proteins with one feature were kept in analysis. Extracted intensities were log2 transformed and quantile normalized. Protein quantitation across conditions was performed pairwise exploiting groupComparison function. Benjamini-Hochberg method was used to adjust p-values.

*ggplot2 3.3.6*^66^, *EnhancedVolcano 1.10.0*,* heatmaply 1.4.*2^67^*and PCA tools 2.4.0*^68^.

R packages were used to plot volcano plots, heatmaps, and PCA. Inkscape 1.2 and Gimp 2.10.32 were used to process the graphics to final panel plots and to generate svg images.

### RT-qPCR

Total RNA was extracted from cells using TRI-Reagent (Sigma-Aldrich, St. Louis, MO) and concentration was determined using nanodrop. M-MLV Reverse Transcriptase (Sigma-Aldrich, St. Louis, MO, USA) was used to reverse transcribe total RNA, and Luna MasterMix (New England Biolabs, UK) was used for quantitative PCR. *HPRT1* and *GAPDH* served as parallel endogenous controls. The data represent means of three technical triplicates within each independent biological replicate (*n* = 3). The primers are listed in Supplementary Table S1 in the supplementary information. The relative mRNA expression levels of each gene were calculated using the 2^−ΔΔCT^ method.

### Western blot

Cells were lysed in NET lysis buffer (120 mM NaCl, 50 mM Tris–HCl [pH 7.2], 1% NP-40 [v/v], 1 mM EDTA, 6 mM EGTA, 6 mg/ml sodium pyrophosphate, 1× protease inhibitor cocktail, and 1× phosphatase inhibitor cocktail [both Sigma–Aldrich]) for 30 min on ice. Lysates were cleared by centrifugation at 13,000 rpm, 30 min, 4 °C and the concentration of proteins was measured by Bradford reagent (Bio-Rad). Proteins were separated by MOPS SDS-PAGE and transferred to nitrocellulose membrane using the Tetra Cell-Blot (Bio-Rad) in Blotting buffer (20 mM Tris, 150 mM glycine, 20% methanol, and pH 8.3). Membranes were blocked in 5% skimmed milk or 3% BSA in PBS with 0. 1% Tween and incubated with primary antibodies Filamin A (FLNA) (Invitrogen MA5-11705), macroD1 (MACD1) (Novus, NBP2-852), Anti-Pyruvate Dehydrogenase E2 (ODP2) (Abcam ab172617), COG3 (Proteintech 11130-I-AP), NPM3 (Proteintech 11960-I-AP), AGR2 K31 (in-house), GAPDH (Abcam ab110305), PD-L1 (Cell Signaling CS13684S), c-myc (Cell Signaling CS5605S), Phospho-eIF2α (Ser51) (Cell Signaling CS3597S), eIF2α (Cell Signaling CS5324S), ZEB1 (Abcam ab203829) at 4 °C overnight. The following day, membranes were washed 4 times with PBS 0.1% Tween, incubated with species-specific secondary horseradish peroxidase-coupled antibodies Peroxidase AffiniPure™ Goat Anti-Rabbit IgG (H + L) (Jackson ImmunoResearch 111-035-003) or Peroxidase AffiniPure™ Goat Anti-Mouse IgG (H + L) (Jackson ImmunoResearch 115-035-003) and then washed again. Signals were revealed using ECL system (solution A: 200 mM TRIS pH 9.4; 10 mM luminol, 405 mM p-coumaric acid; 0.5 mM EDTA pH 8.0; solution B: 0.5 mM EDTA, 8 mM sodium perborate tetrahydrate, 50 mM sodium acetate pH 5.0) in Syngene Gbox (Syngene, USA). The densitometry values were quantified using ImageJ software.

### Flow cytometry PD-L1 surface detection

Cells were seeded in a 6-well plate (SW620, 2 × 10^6^ cells per well) or 5 cm dishes (SW480, 2.5 × 10^6^ cells per dish) and, after 8 h, were exposed to the combination of IFN-γ and TNF-α for 16 h at a concentration stated in the cell culture section. Control samples were treated with a corresponding volume of solvent (water). Afterwards, cells were detached with accutase, and 1 × 10^6^ of cells were washed twice with 3% BSA in PBS (BSA-PBS), resuspended in 100 µl of primary antibody anti-PD-L1 (1:50, eBioscience™, #14-5983-82, clone MIH1), diluted in BSA-PBS and incubated at room temperature for 1 h. After incubation, cells were washed twice in BSA-PBS and resuspended in 100 µl secondary antibody (1:250, goat anti-mouse, Abcam, ab150113, conjugated with Alexa Fluor^®^ 488, polyclonal) diluted in BSA-PBS and incubated at room temperature for 1 h in the dark. Afterwards, cells were washed twice with BSA-PBS, resuspended in 100 ul of BSA-PBS, and signals of 10,000 cells were measured (FacsVerse flow cytometer (BD Biosciences) and analysed (BD FACSuiteTM Software). The level of the fluorescent signal represented by median fluorescence intensity was normalised to the level of negative control (relevant samples incubated with secondary antibody only). All experiments were performed in three biological replicates.

### Statistical analysis

The statistical analysis for RT-qPCR, immunoblot densitometry, and flow cytometry was calculated using GraphPad Prism 10. The data passed the Shapiro-Wilk normalcy test. Either unpaired t test or ordinary one-way ANOVA with Sidak multiple comparison test were used. * *P* ≤ 0.05; ** *P* ≤ 0.01; *** *P* ≤ 0.001; **** *P* ≤ 0.0001.

## Electronic supplementary material

Below is the link to the electronic supplementary material.

Supplementary Material 1

## Data Availability

The datasets generated and/or analysed during the current study are available in the PRoteomics IDEntifications Database (PRIDE) with the dataset identifier PXD053085, https://www.ebi.ac.uk/pride/login. Reviewer can access the dataset using the following account details: Username: reviewer_pxd053085@ebi.ac.uk, Password: yspxnnOEnaEI.

## Abbreviations

AcN: Acetonitrile
CK2: Casein kinase
COG3: Component of oligomeric golgi complex 3
CMS: Consensus molecular subtype
CRC: Colorectal carcinoma
dMMR: Mismatch repair deficiency
EMT: Epithelial-mesenchymal transition
ER: Endoplasmic reticulum
ERCYS: Endoplasmic reticulum-to-cytosol signalling
FLNA: Filamin A
FA: Formic acid
FASP: Filter-aided sample preparation
HDAC: Histone deacetylase
IAA: Iodoacetamide
IDA: Information dependent acquisition
IFN-γ: Interferon gamma
IL-10: Interleukin 10
MACD1: macroD1
MDSCs: Myeloid-derived suppressor cells
MS: Mass spectrometry
MSI-H: microsatellite instability-high
NA: Not available/detected
NK cells: Natural killer cells
NPM3: Nucleoplasmin-3
ODP2: E2 component of pyruvate dehydrogenase complex
PD-1: Programmed cell death protein 1
PDI: Protein disulphide isomerase
PD-L1: Programmed death-ligand 1
PRIDE: PRoteomics IDEntifications Database
QC: Quality control
RT: Room temperature
SWATH MS: Sequential windowed acquisition of all theoretical mass spectra
TFA: Trifluoroacetic acid
TNF-α: Tumour necrosis factor alpha
TG-Fβ: Transforming growth factor beta
TMB: Tumour mutational burden
TME: Tumour microenvironment
UTR: Untranslated region
VEGF: Vascular endothelial growth factor
ZEB1: Zinc finger E-box-binding homeobox 1

## References

[1] Robinson, P. J. & Bulleid, N. J. Mechanisms of disulfide bond formation in nascent polypeptides entering the secretory pathway. *Cells* **9**. 10.3390/cells9091994 (2020). 10.3390/cells9091994 PMC7565403 32872499

[2] Beatty, G. L. & Gladney, W. L. Immune escape mechanisms as a guide for cancer immunotherapy. *Clin. Cancer Res. ***21**. 10.1158/1078-0432.CCR-14-1860 (2015). 10.1158/1078-0432.CCR-14-1860 PMC4334715 25501578

[3] Boisteau, E. et al. Anterior gradient proteins in gastrointestinal cancers: From cell biology to pathophysiology. *Oncogene* **41**. 10.1038/s41388-022-02452-1 (2022). 10.1038/s41388-022-02452-1 36068336

[4] Sommerova, L. et al. ZEB1/miR-200c/AGR2: a New Regulatory Loop modulating the epithelial-mesenchymal transition in lung adenocarcinomas. *Cancers* **12**. 10.3390/cancers12061614 (2020). 10.3390/cancers12061614 PMC7352583 32570918

[5] Sommerova, L., Ondrouskova, E., Vojtesek, B. & Hrstka, R. Suppression of AGR2 in a TGF-beta-induced smad regulatory pathway mediates epithelial-mesenchymal transition. *BMC Cancer* **17**, 546. 10.1186/s12885-017-3537-5 (2017). 28810836 10.1186/s12885-017-3537-5 PMC5557473

[6] Delom, F., Nazaraliyev, A. & Fessart, D. The role of protein disulphide isomerase AGR2 in the tumour niche. *Biol. Cell. ***110**. 10.1111/boc.201800024 (2018). 10.1111/boc.201800024 30238476

[7] Sicari, D. et al. Reflux of endoplasmic reticulum proteins to the cytosol inactivates tumor suppressors. *EMBO Rep.* **22**. 10.15252/embr.202051412 (2021). 10.15252/embr.202051412 PMC8724677 33710763

[8] Hrstka, R. et al. AGR2 oncoprotein inhibits p38 MAPK and p53 activation through a DUSP10-mediated regulatory pathway. *Mol. Oncol.* **10**, 652–662. 10.1016/j.molonc.2015.12.003 (2016). 26733232 10.1016/j.molonc.2015.12.003 PMC5423154

[9] Alsereihi, R. et al. Leveraging the role of the metastatic associated protein anterior gradient homologue 2 in unfolded protein degradation: A novel therapeutic biomarker for cancer. *Cancers* **11**, 890. 10.3390/cancers11070890 (2019). 31247903 10.3390/cancers11070890 PMC6678570

[10] Okuwaki, M. et al. Function of homo- and hetero-oligomers of human nucleoplasmin/nucleophosmin family proteins NPM1, NPM2 and NPM3 during sperm chromatin remodeling. *Nucleic Acids Res. ***40**. 10.1093/nar/gks162 (2012). 10.1093/nar/gks162 PMC3367197 22362753

[11] Huang, N., Negi, S., Szebeni, A. & Olson, M. O. J. Protein NPM3 interacts with the multifunctional nucleolar protein B23/nucleophosmin and inhibits ribosome biogenesis. *J. Biol. Chem. ***280**. 10.1074/jbc.M407856200 (2005). 10.1074/jbc.M407856200 15596447

[12] Wang, H. et al. Pumilio1 regulates NPM3/NPM1 axis to promote PD-L1-mediated immune escape in gastric cancer. *Cancer Lett. ***581**. 10.1016/j.canlet.2023.216498 (2024). 10.1016/j.canlet.2023.216498 38029539

[13] Wei, S., Xing, J., Lu, K., Wang, K. & Yu, W. NPM3 as a novel oncogenic factor and poor prognostic marker contributes to cell proliferation and migration in lung adenocarcinoma. *Hereditas*. **160**. 10.1186/s41065-023-00289-6 (2023). 10.1186/s41065-023-00289-6 PMC10230701 37254219

[14] Han, Y., Liu, D. & Li, L. PD-1/PD-L1 pathway: Current researches in cancer. *Am. J. Cancer Res. ***10** (2020). PMC7136921 32266087

[15] El-Sayes, N., Vito, A. & Mossman, K. Tumor heterogeneity: A great barrier in the age of cancer immunotherapy. *Cancers*. **13**. 10.3390/cancers13040806 (2021). 10.3390/cancers13040806 PMC7918981 33671881

[16] Hewitt, R. E. et al. Validation of a model of colon cancer progression. *J. Pathol.* **192**. https://doi.org/10.1002/1096-9896(2000)9999:9999<::AID-PATH775>3.0.CO;2-K (2000). 10.1002/1096-9896(2000)9999:9999<::AID-PATH775>3.0.CO;2-K 11113861

[17] Dumartin, L. et al. ER stress protein AGR2 precedes and is involved in the regulation of pancreatic cancer initiation. *Oncogene*. **36**, 3094–3103. 10.1038/onc.2016.459 (2017). 27941872 10.1038/onc.2016.459 PMC5467015

[18] Rouillard, A. D. et al. The harmonizome: A collection of processed datasets gathered to serve and mine knowledge about genes and proteins. *Database J. Biol. Databases Curation*10.1093/database/baw100 (2016). 10.1093/database/baw100 PMC4930834 27374120

[19] Ciribilli, Y., Singh, P., Inga, A. & Borlak, J. c-Myc targeted regulators of cell metabolism in a transgenic mouse model of papillary lung adenocarcinoma. *Oncotarget* **7**. 10.18632/oncotarget.11804 (2016). 10.18632/oncotarget.11804 PMC5323172 27602772

[20] Adomavicius, T. et al. The structural basis of translational control by eIF2 phosphorylation. *Nat. Commun. ***10**. 10.1038/s41467-019-10167-3 (2019). 10.1038/s41467-019-10167-3 PMC6513899 31086188

[21] Costa-Mattioli, M. & Walter, P. The integrated stress response: From mechanism to disease. *Science* **368**. 10.1126/science.aat5314 (2020). 10.1126/science.aat5314 PMC8997189 32327570

[22] Bouchalova, P. et al. Characterization of the AGR2 interactome uncovers new players of protein disulfide Isomerase Network in Cancer cells. *MCP* **21**. 10.1016/j.mcpro.2021.100188 (2021). 10.1016/j.mcpro.2021.100188 PMC8816719 34929376

[23] Higa, A. et al. Role of pro-oncogenic protein disulfide isomerase (PDI) family member anterior gradient 2 (AGR2) in the control of endoplasmic reticulum homeostasis. *J. Biol. Chem.* **286**, 44855–44868. 10.1074/jbc.M111.275529 (2011). 22025610 10.1074/jbc.M111.275529 PMC3248018

[24] Garcia-Diaz, A. et al. Interferon Receptor Signaling Pathways Regulating PD-L1 and PD-L2 Expression. *Cell. Rep.* **19**. 10.1016/j.celrep.2017.04.031 (2017). 10.1016/j.celrep.2017.04.031 PMC6420824 28494868

[25] Antonangeli, F. et al. Regulation of PD-L1 expression by NF-κB in Cancer. *Front. Immunol.* **11**, 584626. 10.3389/fimmu.2020.584626 (2020). 33324403 10.3389/fimmu.2020.584626 PMC7724774

[26] Cerami, E. et al. The cBio cancer genomics portal: an open platform for exploring multidimensional cancer genomics data. *Cancer Discov*. **2**. 10.1158/2159-8290.CD-12-0095 (2012). 10.1158/2159-8290.CD-12-0095 PMC3956037 22588877

[27] de Bruijn, I. et al. Analysis and visualization of longitudinal genomic and clinical data from the AACR Project GENIE Biopharma Collaborative in cBioPortal. *Cancer Res. ***83**. 10.1158/0008-5472.CAN-23-0816 (2023). 10.1158/0008-5472.CAN-23-0816 PMC10690089 37668528

[28] Gao, J. et al. Integrative analysis of complex cancer genomics and clinical profiles using the cBioPortal. *Sci. Signal* **6**. 10.1126/scisignal.2004088 (2013). 10.1126/scisignal.2004088 PMC4160307 23550210

[29] Muzny, D. M. et al. Comprehensive molecular characterization of human colon and rectal cancer. *Nature* **487**. 10.1038/nature11252 (2012). 10.1038/nature11252 PMC3401966 22810696

[30] Roelands, J. et al. An integrated tumor, immune and microbiome atlas of colon cancer. *Nat. Med. ***29**. 10.1038/s41591-023-02324-5 (2023). 10.1038/s41591-023-02324-5 PMC10202816 37202560

[31] Vanderlaag, K. E. et al. Anterior gradient-2 plays a critical role in breast cancer cell growth and survival by modulating cyclin D1, estrogen receptor-alpha and survivin. *Breast Cancer Res.* **12**, R32. 10.1186/bcr2586 (2010). 20525379 10.1186/bcr2586 PMC2917027

[32] Gadad, S. S., Shandilya, J., Kishore, A. H. & Kundu, T. K. NPM3, a member of the nucleophosmin/nucleoplasmin family, enhances activator-dependent transcription. *Biochemistry* **49**. 10.1021/bi9021632 (2010). 10.1021/bi9021632 20073534

[33] Wei, Q. et al. Pan-cancer analysis of the prognostic and immunological role of nucleophosmin/nucleoplasmin 3 (NPM3) and its potential significance in lung adenocarcinoma. *CPT* **1**, 10.1016/j.cpt.2023.06.004 (2023). 10.1016/j.cpt.2023.06.004 PMC10846304 38327603

[34] Qin, G. et al. NPM1 upregulates the transcription of PD-L1 and suppresses T cell activity in triple-negative breast cancer. *Nat. Commun.* **11**. 10.1038/s41467-020-15364-z (2020). 10.1038/s41467-020-15364-z PMC7125142 32245950

[35] Qin, G. et al. Targeting the NAT10/NPM1 axis abrogates PD-L1 expression and improves the response to immune checkpoint blockade therapy. *Mol. Med.* **30**. 10.1186/s10020-024-00780-4 (2024). 10.1186/s10020-024-00780-4 PMC10799409 38243170

[36] Chen, Z. et al. Interferon-gamma and tumor necrosis factor-alpha synergistically enhance the immunosuppressive capacity of human umbilical-cord-derived mesenchymal stem cells by increasing PD-L1 expression. *WJSC* **15**. 10.4252/wjsc.v15.i8.787 (2023). 10.4252/wjsc.v15.i8.787 PMC10494569 37700823

[37] Ohmori, Y., Schreiber, R. D. & Hamilton, T. A. Synergy between interferon-gamma and tumor necrosis factor-alpha in transcriptional activation is mediated by cooperation between signal transducer and activator of transcription 1 and nuclear factor kappaB. *J. Biol. Chem.* **272**. 10.1074/jbc.272.23.14899 (1997). 10.1074/jbc.272.23.14899 9169460

[38] Borgo, C., D’Amore, C., Sarno, S., Salvi, M. & Ruzzene, M. Protein kinase CK2: A potential therapeutic target for diverse human diseases. *Signal. Transduct. Target. Ther.* **6**, 183. 10.1038/s41392-021-00567-7 (2021). 33994545 10.1038/s41392-021-00567-7 PMC8126563

[39] de Bie, P. et al. Characterization of COMMD protein-protein interactions in NF-kappaB signalling. *Biochem. J.* **398**, 63–71. 10.1042/bj20051664 (2006). 16573520 10.1042/BJ20051664 PMC1525016

[40] Okazaki, T. & Honjo, T. The PD-1-PD-L pathway in immunological tolerance. *Trends Immunol.* **27**. 10.1016/j.it.2006.02.001 (2006). 10.1016/j.it.2006.02.001 16500147

[41] Dong, Y., Sun, Q. & Zhang, X. PD-1 and its ligands are important immune checkpoints in cancer. *Oncotarget* **8**. 10.18632/oncotarget.13895 (2017). 10.18632/oncotarget.13895 PMC5356790 27974689

[42] Li, Y. et al. Efficacy and safety of anti-PD-1/PD-L1 therapy in the treatment of advanced colorectal cancer: A meta-analysis. *BMC Gastroenterol. ***22**. 10.1186/s12876-022-02511-7 (2022). 10.1186/s12876-022-02511-7 PMC9549670 36217119

[43] Lin, K. X. et al. PD-1 and PD-L1 inhibitors in cold colorectal cancer: Challenges and strategies. *Cancer Immunol. Immunother* **72**. 10.1007/s00262-023-03520-5 (2023). 10.1007/s00262-023-03520-5 PMC10700246 37831146

[44] Rosenbaum, M. W., Bledsoe, J. R., Morales-Oyarvide, V., Huynh, T. G. & Mino-Kenudson, M. PD-L1 expression in colorectal cancer is associated with microsatellite instability, BRAF mutation, medullary morphology and cytotoxic tumor-infiltrating lymphocytes. *Mod. Pathol.* **29**. 10.1038/modpathol.2016.95 (2016). 10.1038/modpathol.2016.95 27198569

[45] Williams, D. S. et al. Nonsense mediated decay resistant mutations are a source of expressed mutant proteins in colon cancer cell lines with microsatellite instability. *PLoS ONE* **5**. 10.1371/journal.pone.0016012 (2010). 10.1371/journal.pone.0016012 PMC3013145 21209843

[46] Chevet, E. et al. AGR2 protein expression in colorectal tumour epithelialcompartment. *Gut* **72**, 2385–2386. 10.1136/gutjnl-2022-328739 (2022). 36591613 10.1136/gutjnl-2022-328739 PMC10715535

[47] Fessart, D. et al. Anterior Gradient-2 (AGR2) is overexpressed in colon cancer and is a potential biomarker of microsatellite instability (MSI) tumors. *bioRxiv*, 2021.2009.2007.459258 (2022). 10.1101/2021.09.07.459258

[48] Boland, C. R. & Goel, A. Microsatellite instability in colorectal cancer. *Gastroenterology ***138**. 10.1053/j.gastro.2009.12.064 (2010). 10.1053/j.gastro.2009.12.064 PMC3037515 20420947

[49] Zheng, Z. et al. T cells in colorectal cancer: Unravelling the function of different T cell subsets in the tumor microenvironment. *IJMS* **24**. 10.3390/ijms241411673 (2023). 10.3390/ijms241411673 PMC10380781 37511431

[50] Berg, K. C. G. et al. Multi-omics of 34 colorectal cancer cell lines—A resource for biomedical studies. *Mol. Cancer* **16**, 116. 10.1186/s12943-017-0691-y (2017). 28683746 10.1186/s12943-017-0691-y PMC5498998

[51] Cai, L., Chen, A. & Tang, D. A new strategy for immunotherapy of microsatellite-stable (MSS)-type advanced colorectal cancer: Multi-pathway combination therapy with PD-1/PD-L1 inhibitors. *Immunology*10.1111/imm.13785 (2024). 38517066 10.1111/imm.13785

[52] Ohm, J. E. et al. VEGF inhibits T-cell development and may contribute to tumor-induced immune suppression. *Blood*. **101**. 10.1182/blood-2002-07-1956 (2003). 10.1182/blood-2002-07-1956 12586633

[53] Sullivan, K. M. et al. Blockade of interleukin 10 potentiates antitumour immune function in human colorectal cancer liver metastases. *Gut* **72**. 10.1136/gutjnl-2021-325808 (2023). 10.1136/gutjnl-2021-325808 PMC9872249 35705369

[54] Tauriello, D. V. F. et al. TGFβ drives immune evasion in genetically reconstituted colon cancer metastasis. *Nature* **554**. 10.1038/nature25492 (2018). 10.1038/nature25492 29443964

[55] Wang, F. et al. Combined anti-PD-1, HDAC inhibitor and anti-VEGF for MSS/pMMR colorectal cancer: A randomized phase 2 trial. *Nat. Med. ***30**. 10.1038/s41591-024-02813-1 (2024). 10.1038/s41591-024-02813-1 38438735

[56] Zhang, Q. et al. Control of cyclin D1 and breast tumorigenesis by the EglN2 prolyl hydroxylase. *Cancer Cell ***16**. 10.1016/j.ccr.2009.09.029 (2009). 10.1016/j.ccr.2009.09.029 PMC2788761 19878873

[57] Martisova, A. et al. AGR2 silencing contributes to metformin-dependent sensitization of colorectal cancer cells to chemotherapy. *Oncol. Lett.* **18**, 4964–4973. 10.3892/ol.2019.10800 (2019). 31612008 10.3892/ol.2019.10800 PMC6781747

[58] Wiśniewski, J. R., Zougman, A., Nagaraj, N. & Mann, M. Universal sample preparation method for proteome analysis. *Nat. Methods* **6**. 10.1038/nmeth.1322 (2009). 10.1038/nmeth.1322 19377485

[59] Bouchal, P. et al. Biomarker discovery in low-grade breast cancer using isobaric stable isotope tags and two-dimensional liquid chromatography-tandem mass spectrometry (iTRAQ-2DLC-MS/MS) based quantitative proteomic analysis. *J. Proteome Res.* **8**, 362–373. 10.1021/pr800622b (2009). 19053527 10.1021/pr800622b

[60] Faktor, J. & Bouchal, P. Building mass spectrometry spectral libraries of human cancer cell lines. *Klin. Onkol* **29**(Suppl 4), 54–58 (2016). 27846721

[61] Bouchal, P. et al. Breast cancer classification based on proteotypes obtained by SWATH mass spectrometry. *Cell. Rep.* **28**, 832-843e837. 10.1016/j.celrep.2019.06.046 (2019). 31315058 10.1016/j.celrep.2019.06.046 PMC6656695

[62] Kong, A. T., Leprevost, F. V., Avtonomov, D. M., Mellacheruvu, D. & Nesvizhskii, A. I. MSFragger: Ultrafast and comprehensive peptide identification in mass spectrometry-based proteomics. *Nat. Methods* **14**, 513–520. 10.1038/nmeth.4256 (2017). 28394336 10.1038/nmeth.4256 PMC5409104

[63] MacLean, B. et al. Skyline: An open source document editor for creating and analyzing targeted proteomics experiments. *Bioinformatics* **26**, 966–968. 10.1093/bioinformatics/btq054 (2010). 20147306 10.1093/bioinformatics/btq054 PMC2844992

[64] Reiter, L. et al. mProphet: Automated data processing and statistical validation for large-scale SRM experiments. *Nat. Methods* **8**, 430–435. 10.1038/nmeth.1584 (2011). 21423193 10.1038/nmeth.1584

[65] Choi, M. et al. MSstats: An R package for statistical analysis of quantitative mass spectrometry-based proteomic experiments. *Bioinformatics* **30**, 2524–2526. 10.1093/bioinformatics/btu305 (2014). 24794931 10.1093/bioinformatics/btu305

[66] Villanueva, R. A. M. & Chen, Z. J. ggplot2: Elegant graphics for data analysis (2nd ed.). *Meas-Interdiscip. Res.* **17**, 160–167. 10.1080/15366367.2019.1565254 (2019).

[67] Galili, T., O’Callaghan, A., Sidi, J. & Sievert, C. Heatmaply: An R package for creating interactive cluster heatmaps for online publishing. *Bioinformatics* **34**, 1600–1602. 10.1093/bioinformatics/btx657 (2018). 29069305 10.1093/bioinformatics/btx657 PMC5925766

[68] PCAtools, P. C. A. Everything Principal Components Analysis. R package version 2.16.0 (2024). https://github.com/kevinblighe/PCAtools.

