# Supplementary material for: Characterization of the AGR2-NPM3 axis uncovers the AGR2 involvement in PD-L1 regulation in colorectal cancer
Source: Sci Rep. 2024 Sep 20;14:21926. doi: 10.1038/s41598-024-72990-z (PMC11413233; doi:10.1038/s41598-024-72990-z)
Supplement: Supplementary file 1 — Supplementary Material 1 [file 41598_2024_72990_MOESM1_ESM.pdf]

Supplementary Information:

Characterisation of the AGR2-NPM3 axis uncovers the AGR2 involvement in PD-L1 regulation in colorectal cancer.

Andrea Martišová[1, 2#], Jakub Faktor[3#], Tereza Sosolikova[1, 4], Iveta Klemesova[1], Tamara Kolarova[1], Jitka Holcakova[1], Roman Hrstka[1]*

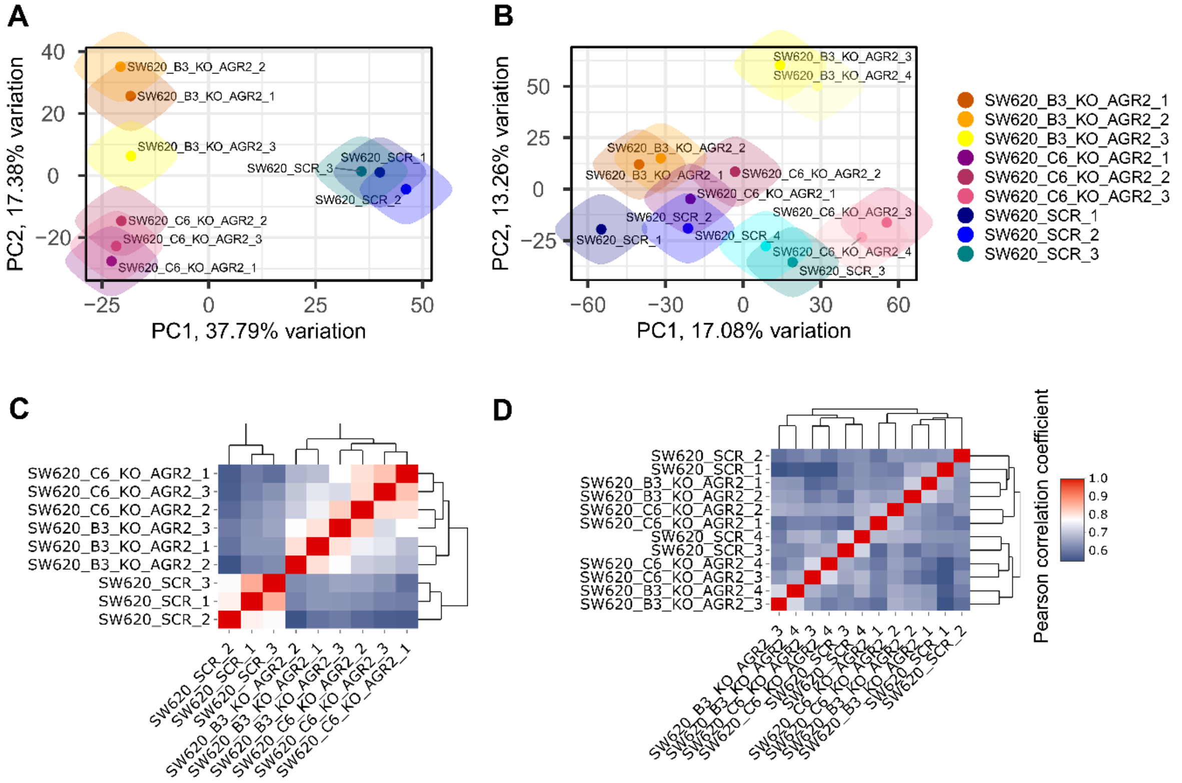

Figure S1: Mass spectrometry diagnostic plots and clustering. Diagnostic graphs for two independent SWATH MS datasets (Exp 1 and Exp 2) comparing SW620 scr (negative control endogenously expressing AGR2) with two AGR2 CRISPR-Cas9 mediated knockout clones, SW620 KOAGR2 B3 and SW620 KOAGR2 C6. (**A**) shows PCA analysis of samples from dataset 1 (Exp 1) while (**B**) shows PCA analysis of samples from dataset 2 (Exp 2). (**C**) Sample correlation heatmap (Pearson correlation) of samples from dataset 1 (Exp 1) and (**D**) sample correlation heatmap (Pearson correlation) of samples from dataset 2 (Exp 2).

Supplementary Data 1. Full lists of quantitated proteins from three SWATH MS datasets (uploaded as independent xls file). First two sheets contain comparison of SW620 scr (negative control endogenously expressing AGR2) with two AGR2 CRISPR-Cas9 mediated knockout clones (Exp 1 and Exp 2), and the third one shows comparison of AGR2 overexpressing SW480 clone to control cells (SW480 pcDNA3) (Exp 3). Data were analysed using two SWATH MS data analysis pipelines: one with ProteinPilot for protein identification and MicroSWATH for data extraction with statistical analysis in MarkerView plugin, and the other with MSFragger

for protein identification and Skyline for data extraction with statistical analysis in MSstats R package.

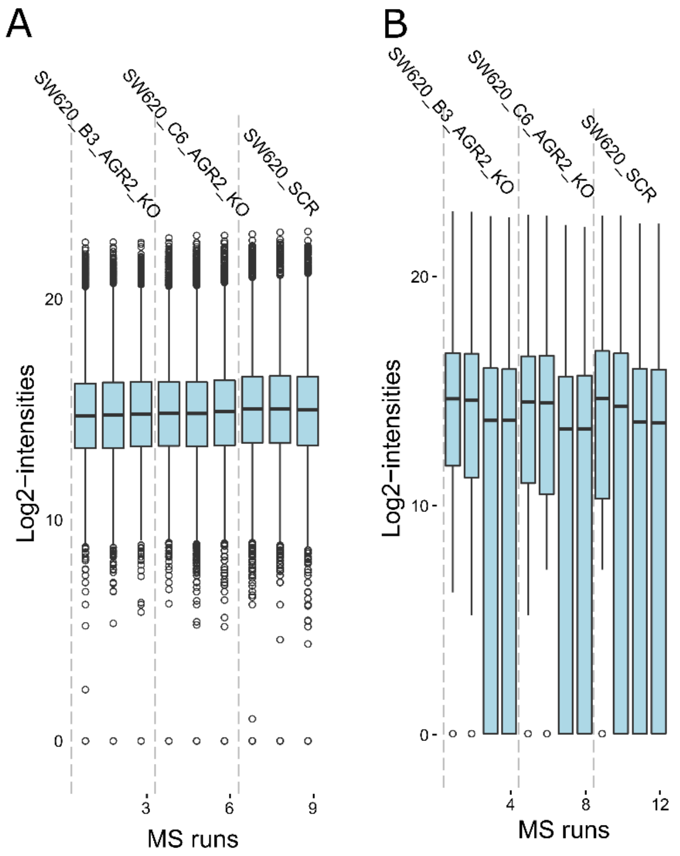

Figure S2: Log intensity plot for proteins quantitated in SW620 B3, C6 and SCR proteotypes. (**A**) Protein intensity bar plot in EXP1 showing high consistency in protein intensities within replicates and also conditions suggesting successful sample preparation. (**B**) Protein intensity bar plot in EXP2 showing slightly compromised consistency within protein intensity profiles across replicates and conditions suggesting compromised sample preparation if compared to A. A and B suggests that dataset A should be used as main dataset and B should be used as auxiliary dataset.

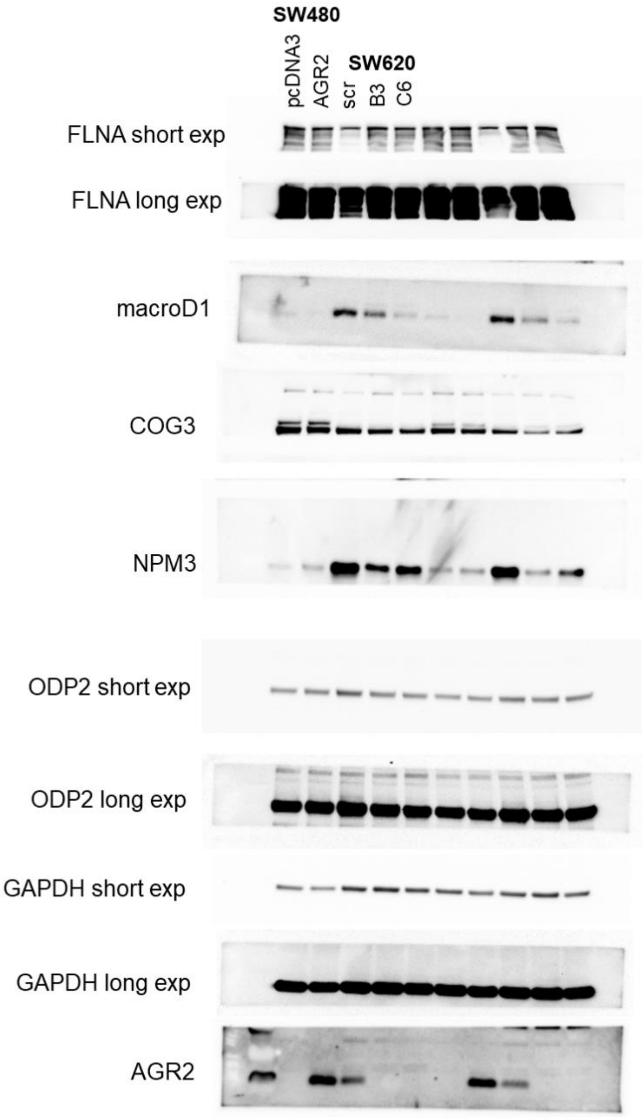

Figure S3: Uncropped western blot membranes from Fig. 2.

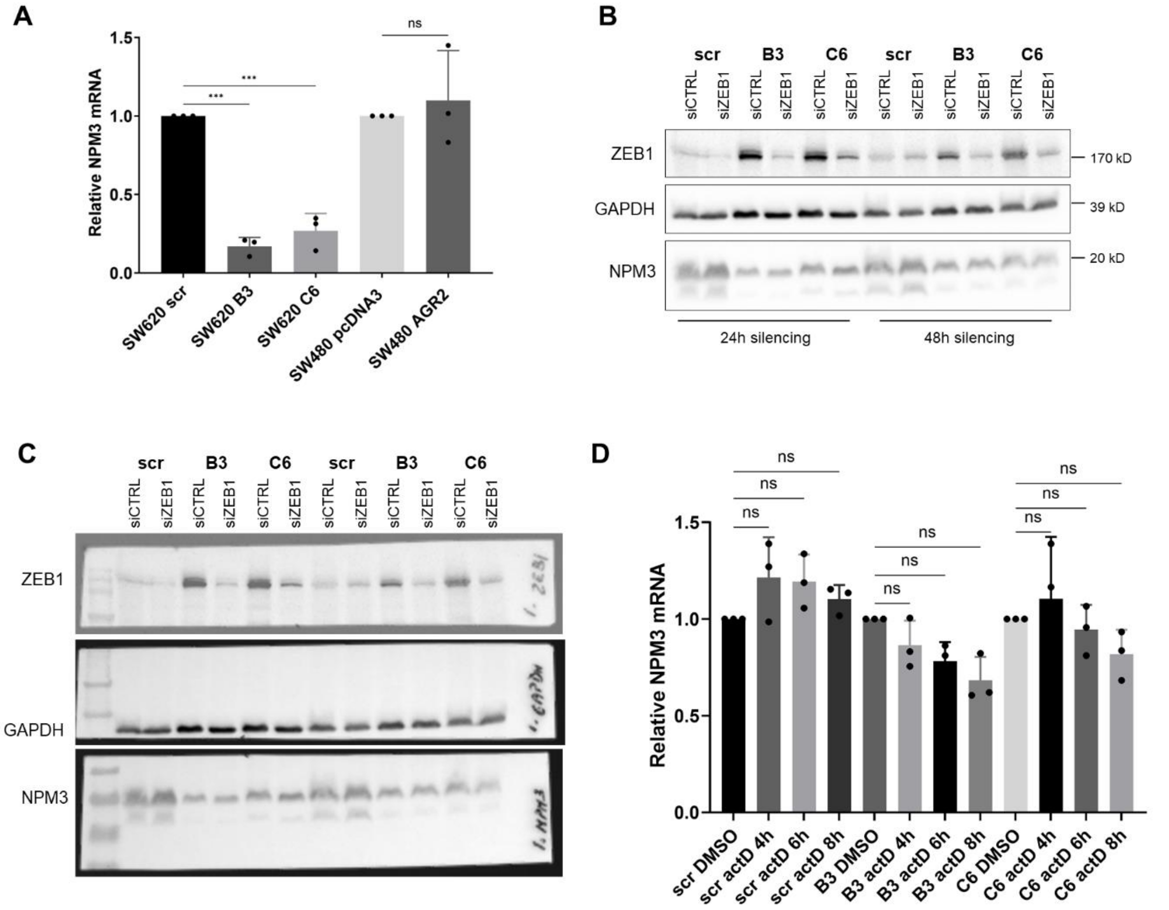

Figure S4: Possible regulatory mechanisms of NPM3 by AGR2. (**A**) Analysis of NPM3 mRNA levels in SW620 and SW480 cell lines and their respective AGR2 clones. RT-qPCR was performed in three biological replicates, and the data are represented as 2^{-ddCt} mean values ± SD. HPRT1 served as endogenous control. (**B**) Western blot analysis of NPM3 levels after cells were silenced with siRNA against ZEB1 for 24 and 48h. (**C**) Corresponding uncropped membranes for siZEB1 silencing. (**D**) RT-qPCR analysis of NPM3 expression in SW620 scr and AGR2 knockout clones B3 and C6 after actinomycin D treatment for 2, 4, and 6 hours. Data are presented as mean ± SD. GAPDH served as endogenous control.

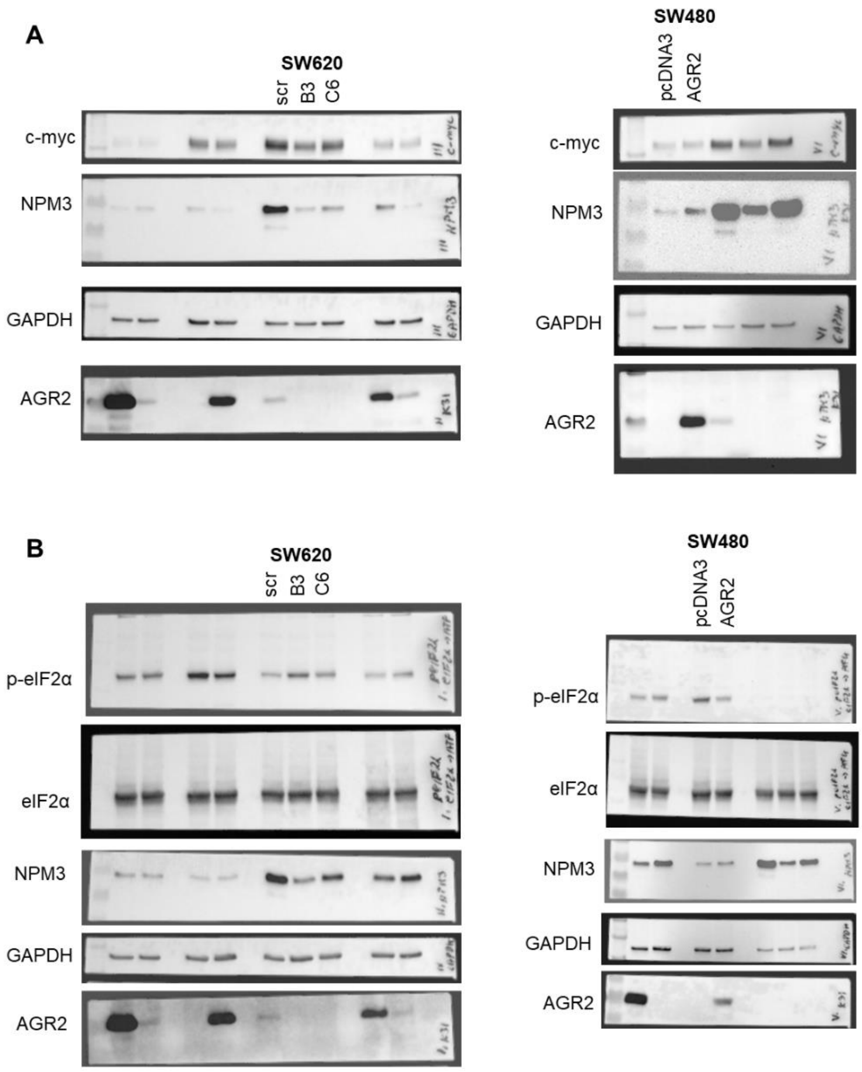

Figure S5: Uncropped western blot membranes from (**A**) Fig. 3 B, and (**B**) Fig. 3 C.

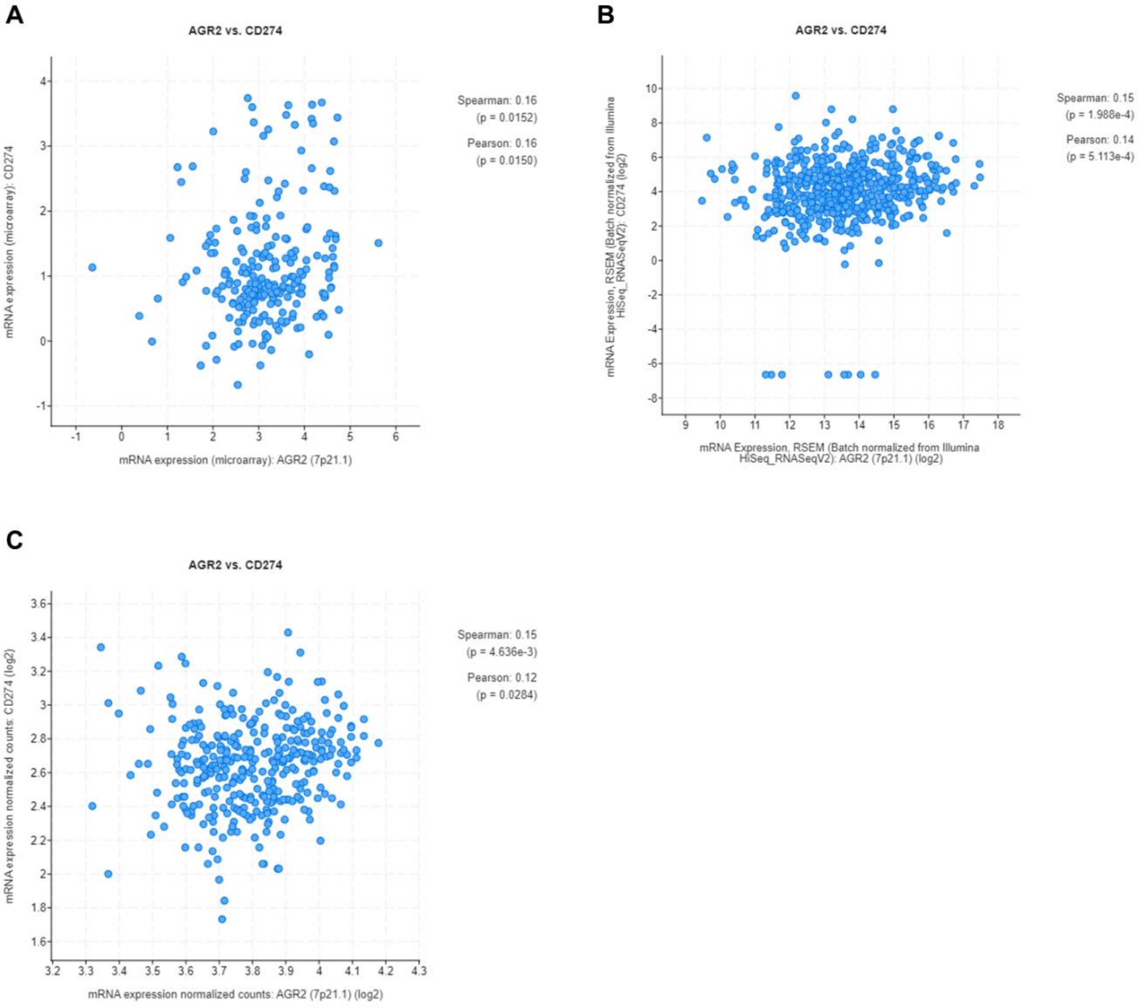

Figure S6: Correlation analysis between AGR2 and CD247/PD-L1 from cBioPortal database. Significant correlations based on (**A**) GDAC Firehose dataset (previously known as TCGA provisional), (**B**) Colorectal Adenocarcinoma TCGA PanCancer Atlas consisting of 526 samples/patients, (**C**) dataset generated by whole exome and transcriptome sequencing of 348 Colon Cancers.

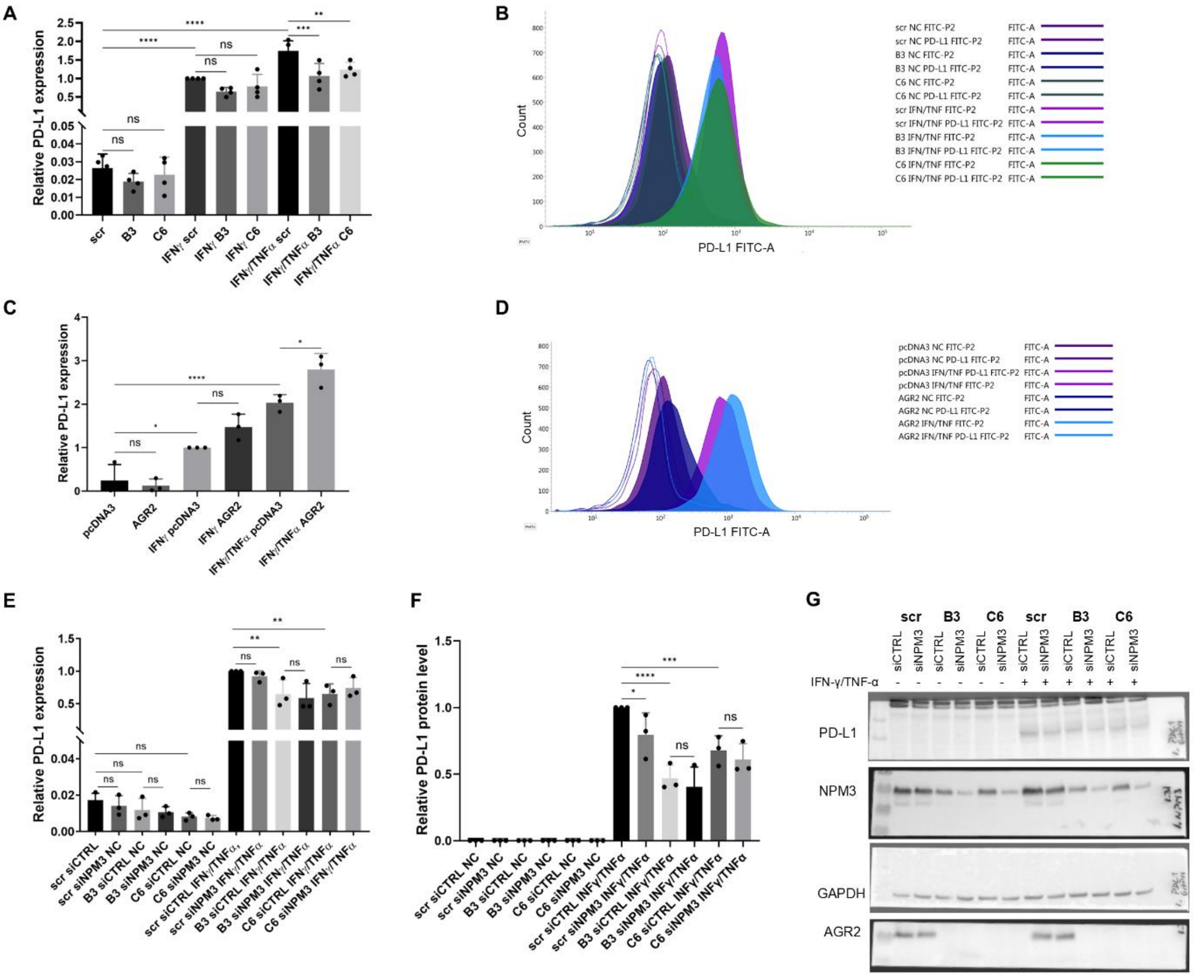

Figure S7: AGR2-dependent changes in PD-L1. (**A**) RT-qPCR analysis of PD-L1 mRNA levels in SW620 scr and its two AGR2 knockout clones B3 and C6, before and after induction with INF-γ or its combination with TNF-α after 4h treatment. Values are normalised to scr IFN- γ and represent mean ± SD of four biological replicates. HPRT1 served as endogenous control. (**B**) Histrograms showing surface level of PD-L1 analysed by flow cytometry in SW620 cell line and its respective KOAGR2 clones. Line histograms represent negative controls while filled histograms are samples labelled with anti-PD-L1 antibody. (**C**) PD-L1 mRNA levels in SW480 pcDNA3 and AGR2 with INF- γ and INF- γ/TNF-α after 4h. Values are normalised to pcDNA3 INF- γ and represent mean ± SD of three independent replicates. HPRT1 served as endogenous control. (**D**) Histrograms showing surface level of PD-L1 analysed by flow cytometry in SW480 cell line and its respective AGR2 clone. Line histograms represent negative controls while filled histograms are samples labelled with anti-PD-L1 antibody. (**E**) Analysis of PD-L1 mRNA levels in SW620 cell line and the respective AGR2 clones. RT-qPCR was performed in three biological replicates, and the data are represented as 2^{-ddCt} mean values ± SD. HPRT1 served as endogenous control. (**F**) Graph showing the mean ± SD densitometry values of PD-L WB analysis of three independent biological replicates normalised to SW620 scr siCTRL cells for NC and INF- γ/TNF-α treatment. GAPDH served as a loading control. (**G**) Uncropped western blot membranes for Fig. 4 F.

Table S1: Sequences of RT-qPCR primers

Target	Forward	Reverse
GAPDH	5´-GAAGGTGAAGGTCGGAGTC-3´	5´-GAAGATGGTGATGGGATTTC-3´
HPRT1	5´-ACAAGTTTGTTGTAGGATATGCCC-3´	5´-GGCGATGTCAATAGGACTCCA-3´
NPM3	5´-GATGACTTCCAGCTCCAACCA-3´	5´-CTCGCTCTCCTCCTCAGAAACA-3´
PD-L1	5´-TGCCGACTACAAGCGAATTACTG-3´	5´-CTGCTTGTCCAGATGACTTCGG-3´
